# Synergistic Interactions Between Medicinal Plant Bioactive and Standard Chemotherapy in Gastric Cancer: Preclinical Evidence and Translational Pitfalls

**DOI:** 10.3390/biomedicines14040947

**Published:** 2026-04-21

**Authors:** Emilia Daliana Muntean, Daniela-Cornelia Lazăr, Ana-Maria Pah, Christian Banciu, Sorin-Dan Chiriac, Iasmina Denisa Boantă, Florin Muntean, Iulian-Alexandru Blidişel, George-Andrei Drăghici, Radu Jipa

**Affiliations:** 1 University Clinic of Internal Medicine IV, Faculty of Medicine, “Victor Babeş” University of Medicine and Pharmacy, 2 Eftimie Murgu Square, 300041 Timisoara, Romania; emilia.muntean@umft.ro (E.D.M.); lazar.daniela@umft.ro (D.-C.L.); iasmina.boanta@rezident.umft.ro (I.D.B.); 2 Doctoral School, Faculty of Medicine, “Victor Babeş” University of Medicine and Pharmacy, 2 Eftimie Murgu Square, 300041 Timisoara, Romania; 3 University Clinic for Outpatient Internal Medicine, Cardiovascular Prevention, and Rehabilitation, Faculty of Medicine, “Victor Babeş” University of Medicine and Pharmacy, 2 Eftimie Murgu Square, 300041 Timisoara, Romania; 4 University Clinic of Surgery III, Faculty of Medicine, “Victor Babeş” University of Medicine and Pharmacy, 2 Eftimie Murgu Square, 300041 Timisoara, Romania; chiriac.sorin@umft.ro (S.-D.C.); munteanu.florin@umft.ro (F.M.); 5 Department of Surgical Semiology I and Thoracic Surgery, Faculty of Medicine, “Victor Babeş” University of Medicine and Pharmacy, 2 Eftimie Murgu Square, 300041 Timisoara, Romania; blidy@umft.ro; 6 Center for Hepato-Biliary and Pancreatic Surgery, “Victor Babeş” University of Medicine and Pharmacy, 2 Eftimie Murgu Square, 300041 Timisoara, Romania; 7 University Clinic of Toxicology, Drug Industry, Management, Marketing and Dermatopharmacy, Faculty of Pharmacy, “Victor Babeş” University of Medicine and Pharmacy, 2 Eftimie Murgu Square, 300041 Timisoara, Romania; draghici.george-andrei@umft.ro; 8 Research Center for Pharmaco-Toxicological Evaluations, “Victor Babeş” University of Medicine and Pharmacy, 2 Eftimie Murgu Square, 300041 Timisoara, Romania; 9 Department of “Life Science”, Faculty of Medicine, “Vasile Goldiş” Western University of Arad, 310048 Arad, Romania; jipa.radu@uvvg.ro

**Keywords:** gastric cancer, medicinal plants, phytocompounds, chemosensitization

## Abstract

Gastric cancer remains a highly heterogeneous malignancy in which chemotherapy response is limited by intrinsic and acquired resistance, cumulative toxicity, and the restricted predictive value of conventional preclinical models. This review critically synthesizes evidence on selected medicinal plants and their bioactive phytocompounds as adjuncts to standard chemotherapy for gastric cancer, with an emphasis on mechanistic plausibility, preclinical synergy, and translational barriers. Across the reviewed literature, phytocompounds from *Curcuma longa*, *Scutellaria baicalensis*, *Camellia sinensis*, *Syzygium aromaticum*, *Glycyrrhiza glabra*, *Allium sativum*, *Marsdenia tenacissima*, and *Rhus verniciflua* showed anticancer or chemopreventive activity through multitarget effects on apoptosis, proliferation, invasion, inflammation, oxidative stress, and resistance-associated signaling. The most convincing chemosensitizing evidence involved curcumin, wogonin, baicalein, EGCG, which enhanced the activity of fluoropyrimidines, platinum agents, paclitaxel, doxorubicin, or related antitumor regimens in selected gastric cancer models. However, the evidence base remains heterogeneous and is constrained by variable extract standardization, incomplete dose reporting, poor bioavailability, insufficient pharmacokinetic/pharmacodynamic integration, and underuse of clinically relevant model systems. Overall, medicinal plant bioactives remain promising adjunct candidates in gastric cancer. Still, meaningful translation will require chemically defined interventions, rigorous synergy analysis, interaction-aware study design, and validation in advanced preclinical and clinical settings.

## 1. Introduction

Gastric cancer (GC) remains a major global health burden, ranking among the most commonly diagnosed malignancies and the leading causes of cancer-related mortality worldwide [1,2]. Despite advances in surgery, systemic chemotherapy, targeted therapies, and immunotherapy, the prognosis for patients with advanced or metastatic disease remains poor [3]. A major obstacle to therapeutic progress is the marked molecular, histological, and cellular heterogeneity of GC, which contributes to variable drug sensitivity, intrinsic and acquired chemoresistance, and disease recurrence [1,3,4]. In addition, the gastric tumor microenvironment, composed of cancer stem cells, cancer-associated fibroblasts, immune cells, and extracellular matrix components, profoundly influences therapeutic response and may promote treatment failure [1,5,6].

Current systemic treatment for advanced GC is largely based on fluoropyrimidine- and platinum-containing regimens, supplemented in selected settings with targeted agents such as trastuzumab and ramucirumab, and, more recently, with immune checkpoint inhibitors targeting the PD-1/PD-L1 axis [3,4,7]. Perioperative and neoadjuvant chemotherapy have also become integral components of treatment for locally advanced resectable disease [4]. Nevertheless, overall clinical benefit remains limited by dose-limiting toxicities, narrow therapeutic windows, and the frequent emergence of resistance [4,8]. Molecularly informed clinical studies, including umbrella-type therapeutic stratification efforts, have further underscored the profound impact of interpatient heterogeneity on treatment response and the limited efficacy gains achieved by empiric treatment strategies [3]. Collectively, these limitations justify the search for adjunctive approaches that can potentiate chemotherapy, improve tolerability, and delay the development of resistance.

In this context, medicinal plants and their bioactive constituents have attracted growing attention as potential adjuvants in cancer therapy. Interest in plant-derived compounds is supported not only by their historical use in medicine but also by the central role of natural products in anticancer drug development, as exemplified by vincristine, paclitaxel, and camptothecin derivatives [2,9]. Beyond these established agents, a broad range of phytochemicals, including polyphenols, flavonoids, terpenoids, alkaloids, and organosulfur compounds, have shown anticancer activity in preclinical GC models by modulating cell proliferation, apoptosis, invasion, metastasis, and stress-response signaling [2,10]. Their pleiotropic, multitarget mode of action is particularly attractive in GC, where treatment failure usually arises from the interplay of multiple tumor-intrinsic and microenvironment-dependent resistance mechanisms [1,5,6,10].

The possibility that phytochemicals may act synergistically with standard chemotherapeutic agents has emerged as a particularly promising field of investigation. In this setting, synergy refers to a combined effect that exceeds the expected additive activity of the individual agents and may therefore enable dose reduction, enhanced antitumor efficacy, and reduced systemic toxicity. In preclinical GC models, numerous plant-derived bioactives have been reported to enhance the activity of agents such as 5-fluorouracil, cisplatin, oxaliplatin, and doxorubicin through mechanisms including apoptosis induction, inhibition of epithelial-to-mesenchymal transition, suppression of cancer stemness, modulation of oxidative stress, interference with autophagy, and downregulation of multidrug resistance pathways [1,5,9,11,12,13,14,15]. Importantly, however, true pharmacological synergy should be distinguished from simple co-activity or chemosensitization and should ideally be supported by formal quantitative combination analyses. In the present review, we therefore differentiate among three evidence levels: formal synergy demonstrated by quantitative combination analysis, chemosensitization without formal synergy analysis, and general anti-cancer activity without direct evidence of chemotherapy interaction.

Despite the growing body of preclinical evidence, translating these findings into clinically relevant strategies remains limited. A major reason is the limited predictive value of conventional experimental systems. Two-dimensional cell cultures do not adequately recapitulate the three-dimensional architecture, stromal interactions, and immune microenvironment of human tumors, thereby limiting their translational relevance [15,16,17]. More advanced models, including organoids, patient-derived xenografts, assembloids, and bioengineered three-dimensional platforms, offer improved physiological fidelity and may better capture treatment-response heterogeneity [18,19,20,21]. However, their use to evaluate phytochemical–chemotherapy combinations in GC remains relatively underdeveloped. Additional translational barriers include the poor bioavailability and unfavorable pharmacokinetic properties of many phytochemicals, insufficient standardization of natural product preparations, heterogeneity in experimental design, and the paucity of well-designed clinical studies to validate preclinical observations [12,13,22]. These challenges are further compounded by the substantial inter- and intratumoral heterogeneity of GC, which limits biomarker development and complicates rational patient stratification [23,24].

Accordingly, this review offers a focused synthesis of preclinical evidence on interactions between medicinal plant bioactives and standard chemotherapy in GC, with particular emphasis on translational barriers that may limit clinical applicability. Section 1 briefly outlines the review’s scope and the approach to literature selection. Section 2 outlines current chemotherapy standards and the principal unmet clinical needs. Section 3 discusses selected medicinal plants and phytochemicals with documented anticancer activity in GC models. Section 4 examines the available evidence for phytochemical–chemotherapy synergy in preclinical GC systems. Section 5 presents the conclusions. Rather than merely cataloging natural compounds, this review adopts an integrative perspective to evaluate the robustness, mechanistic rationale, and translational plausibility of reported synergy data in the context of GC.

### Review Scope and Literature Selection Approach

This article was designed as a focused narrative review rather than a systematic review or meta-analysis. The literature was identified through targeted searches of PubMed, Scopus, and Google Scholar up to March 2026 using combinations of terms related to gastric cancer (“gastric cancer”, “stomach cancer”, “gastric adenocarcinoma”), medicinal plants and phytocompounds (“medicinal plants”, “phytochemicals”, “plant extract”, “polyphenols”, “flavonoids”, and the names of individual plants or major bioactives), and chemotherapy interaction concepts (“5-fluorouracil”, “cisplatin”, “oxaliplatin”, “paclitaxel”, “docetaxel”, “doxorubicin”, “irinotecan”, “S-1”, “chemotherapy”, “combination”, “chemosensitization”, and “synergy”). No strict start-date restriction was imposed; however, priority was given to more recent literature, while older studies were included when they were considered mechanistically important, historically influential, or repeatedly cited in the gastric-cancer field.

The review was intentionally focused on medicinal plants and phytocompounds with gastric-cancer-specific relevance rather than attempting an exhaustive catalog of all botanical agents with putative anticancer activity. Plants or bioactives were prioritized when they met one or more of the following criteria: (i) direct evidence of interaction with standard chemotherapy in gastric cancer models; (ii) reproducible anticancer or resistance-modifying activity in gastric cancer systems with plausible mechanistic relevance to chemosensitivity; and/or (iii) sufficient recurrence or translational interest in the gastric-cancer literature to justify critical discussion. This approach led to the prioritization of eight medicinal plants for detailed evaluation in the main text.

Evidence strength was judged narratively rather than by formal systematic review scoring tools. Greater weight was given to studies that used chemically defined interventions, clearly reported doses and treatment conditions, evaluated interactions with standard gastric-cancer chemotherapy agents, used gastric-cancer-relevant models, included mechanistic support, and, where applicable, provided formal combination analysis or in vivo validation. Studies based primarily on general anticancer activity, poorly characterized extracts, indirect non-gastric evidence, or limited methodological detail were interpreted more cautiously, especially when translational conclusions might otherwise be overstated.

## 2. Chemotherapy in Gastric Cancer: Current Standards and Unmet Needs

Systemic chemotherapy remains a central component of gastric cancer treatment, particularly for patients with locally advanced, unresectable, or metastatic disease. Although targeted therapies and immune checkpoint inhibitors have broadened the therapeutic landscape for selected molecular subgroups, conventional cytotoxic chemotherapy remains the mainstay of treatment for most patients and, therefore, the relevant clinical reference point for evaluating adjunctive strategies [25,26,27,28,29].

### 2.1. Standard Chemotherapy Backbones Relevant to Phytochemical Combination Studies

Current chemotherapy for gastric cancer is built mainly on a relatively limited group of cytotoxic drug classes that remain central across disease settings, namely fluoropyrimidines, platinum compounds, and taxanes, with irinotecan used in selected settings, particularly after progression on first-line therapy [27,28,30]. Although treatment algorithms have become more complex with the addition of targeted agents and immune checkpoint inhibitors in molecularly selected subgroups, conventional cytotoxic regimens still represent the therapeutic backbone for most patients and therefore remain the most relevant reference point when discussing adjunctive phytochemical strategies [25,26,27,28,29,30,31].

Among these agents, fluoropyrimidines continue to play a foundational role. 5-fluorouracil (5-FU) has long been one of the major active drugs in advanced gastric cancer and remains embedded in multiple combination regimens used historically and in contemporary practice [26,27,32]. Oral fluoropyrimidines such as capecitabine and S-1 were developed to improve treatment convenience and maintain prolonged drug exposure while avoiding some of the practical limitations associated with continuous infusional 5-FU [32,33,34,35]. These agents are now widely incorporated into standard gastric-cancer regimens, particularly in platinum-based doublets, and remain directly relevant to the present review because many of the reported plant-derived chemosensitizing effects in preclinical studies involve modulation of fluoropyrimidine response [27,28,35,36,37,38,39].

Platinum compounds constitute the second major backbone component. Cisplatin has historically been used in several reference regimens. It remains clinically important, but its use is constrained by nephrotoxicity, hydration requirements, and cumulative treatment burden, especially in older or frail patients [28,35,40,41]. Oxaliplatin has therefore become an important alternative in many fluoropyrimidine-based combinations due to its more favorable tolerability profile in some settings. However, it introduces its own dose-limiting toxicities, particularly cumulative peripheral neuropathy [29,38,42,43,44]. From the perspective of adjunctive plant-bioactive research, this distinction is important because many proposed phytochemical combinations are not intended to replace standard platinum-based therapy but rather to increase tumor sensitivity to these regimens or reduce the dose intensity needed to achieve antitumor activity.

Taxanes are the third major class of conventional cytotoxic agents in gastric cancer. Paclitaxel and docetaxel are used across different combinations and treatment lines, including first-line, later-line, and perioperative settings, depending on disease stage, patient fitness, and local practice patterns [27,28,30,45]. Paclitaxel is often administered weekly to improve tolerability while preserving activity. In contrast, docetaxel has also been incorporated into more intensive regimens, including triplet approaches designed to improve efficacy at the cost of greater hematologic toxicity [30,31,40,45,46,47,48]. These agents are highly relevant in the context of phytochemical synergy because several preclinical gastric cancer studies examine whether plant-derived compounds can enhance taxane-induced apoptosis, reverse taxane resistance, or improve responses in models with aggressive or treatment-refractory phenotypes.

Irinotecan occupies a somewhat narrower but still established role, particularly in pretreated or later-line disease, where it may be used after failure of fluoropyrimidine- and platinum-based therapy [25,28,49]. Although less widely used than fluoropyrimidines, platinum agents, or taxanes, it remains relevant because some experimental combination studies evaluate phytochemical interactions not only with first-line backbone agents but also with drugs used in resistant or previously treated disease. More broadly, the availability of later-line cytotoxic options underscores a central clinical reality in gastric cancer: many patients eventually require sequential treatment because durable control with first-line chemotherapy alone is uncommon [25,46,49].

Chemotherapy selection also varies by clinical setting. In advanced or metastatic disease, the primary goal is usually disease control and prolongation of survival. In contrast, in perioperative, neoadjuvant, or adjuvant settings, treatment must be balanced against postoperative recovery, nutritional vulnerability, and overall feasibility of treatment [27,28,50,51,52]. This is particularly important in gastric cancer, where surgery-related physiological stress, altered gastrointestinal function, and host frailty may significantly affect chemotherapy delivery [37,50]. Accordingly, the practical value of any adjunctive strategy cannot be judged solely by whether it enhances in vitro cytotoxicity; it must also be evaluated in light of toxicity constraints and treatment-intensity limits that define real-world chemotherapy use.

Taken together, standard chemotherapy for gastric cancer remains anchored in fluoropyrimidine-, platinum-, and taxane-based regimens, with irinotecan used in selected lines of therapy [27,28,30]. This therapeutic framework is essential for interpreting the preclinical literature on medicinal plant bioactives. In this review, these drug classes are treated as the primary conventional partners in reported phytochemical combinations because they are the agents most commonly used in clinical gastric cancer management and the most frequent comparators in experimental chemosensitization studies.

### 2.2. Major Limitations: Resistance, Dose-Limiting Toxicities, and Other Unmet Needs

Despite the central role of cytotoxic chemotherapy in gastric cancer, its overall clinical benefit remains limited by several persistent problems. First, treatment efficacy is frequently constrained by intrinsic or acquired resistance, and durable disease control remains difficult to achieve in advanced disease [25,28,30,45,49,53,54]. Although objective responses can occur, they are often incomplete and temporary, and many tumors ultimately progress despite sequential lines of therapy. This limited durability is one of the main reasons adjunctive approaches to enhance chemosensitivity have attracted sustained interest.

Second, toxicity remains a major limitation across standard regimens. Myelosuppression is common with fluoropyrimidine-, platinum-, taxane-, and irinotecan-based therapy, whereas cisplatin is additionally limited by nephrotoxicity and hydration burden, and oxaliplatin and taxanes are frequently associated with cumulative neuropathy [28,31,38,40,41,42,43,44,45,48,50,55]. These adverse effects are clinically important not only because they reduce tolerability but also because they lead to dose reduction, treatment delay, or premature discontinuation, thereby affecting the dose intensity actually delivered in practice.

A further challenge is the substantial heterogeneity of gastric cancer at both the tumor and patient level. Older patients, postoperative patients, and individuals with nutritional compromise or significant comorbidity often tolerate treatment poorly, while robust predictive biomarkers for conventional cytotoxic therapy remain limited outside selected targeted settings [25,27,30,37,42,43,50,56]. Consequently, many patients still receive broadly similar chemotherapy backbones despite marked biological and clinical variability in treatment sensitivity and toxicity risk.

These limitations justify exploring adjunctive strategies to enhance antitumor efficacy, modulate resistance-associated pathways, or improve the therapeutic index of standard treatment. In this context, medicinal plant bioactives are of interest not as substitutes for chemotherapy but as candidate chemotherapy-modifying agents, whose value depends on mechanistic plausibility, pharmacologic compatibility, and translational feasibility. This framework is essential for interpreting the following sections, which address medicinal plant phytocompounds in gastric cancer, their reported synergistic interactions with standard chemotherapy, and the main barriers to clinical translation.

## 3. Medicinal Plants and Phytocompounds with Anticancer/Chemopreventive Activity in Gastric Cancer

Medicinal plants and their bioactive constituents have attracted growing attention in gastric cancer research because they may target multiple stages of gastric carcinogenesis, from early chemopreventive events to the suppression of established tumor growth. In this context, chemoprevention generally refers to the use of natural or synthetic agents to prevent, delay, or reverse carcinogenesis. In contrast, anticancer activity more commonly denotes direct inhibitory effects on tumor cell survival, proliferation, invasion, or tumor expansion in established disease models [57,58].

A growing body of evidence indicates that plant extracts and purified phytocompounds, including polyphenols, flavonoids, terpenoids, and alkaloids, can exert antiproliferative, proapoptotic, anti-inflammatory, and anti-invasive effects in GC models through multitarget modulation of signaling networks, such as the NF-κB, MAPK, and JAK/STAT pathways [9,57,58]. However, interpreting this literature requires caution, as “activity in GC” encompasses heterogeneous experimental contexts, including prevention of gastric tumorigenesis, inhibition of tumor growth in animal models, and cytotoxic or chemosensitizing effects in cultured GC cells [58]. These distinctions are translationally important because promising preclinical effects do not necessarily translate into clinical utility, particularly in the presence of limited bioavailability, inconsistent extract standardization, and uncertainty about clinically achievable exposure and long-term safety [58,59,60,61,62,63].

Not all plants discussed in this section are supported by the same depth or type of evidence. Across the reviewed literature, the strongest and most mechanistically developed preclinical evidence for chemotherapy-modifying activity in gastric cancer currently concerns curcumin from Curcuma longa and flavones from Scutellaria baicalensis, especially wogonin and baicalein. Other candidates remain of interest but are supported by narrower, more formulation-dependent, more indirect, or more preliminary evidence. In some cases, the available literature is stronger for general anticancer activity than for direct evidence of a gastric-cancer-specific chemotherapy interaction. This distinction is maintained throughout the following sections to avoid treating all plant-derived candidates as equally substantiated.

### 3.1. Curcuma longa (Turmeric)

#### 3.1.1. Relevant Phytocompounds of *Curcuma longa*

The anticancer and chemopreventive literature on *Curcuma longa* (turmeric), both in general and in the context of gastric cancer, has focused primarily on rhizome-derived curcuminoids, namely curcumin (diferuloylmethane), demethoxycurcumin (DMC), and bisdemethoxycurcumin (BDMC), with curcumin representing the predominant constituent [63,64,65,66,67,68]. Curcuminoids are generally reported to account for only a low single-digit fraction of turmeric preparations, commonly around 3–5% of rhizome-derived material, with curcumin being the major component within this fraction [63,67,68]. Although turmeric is most commonly used as dried rhizome powder, phytochemical studies across different plant parts, including rhizomes, leaves, and flowers, indicate that its biological activity cannot always be attributed to a single purified compound [57,66,67].

Importantly, turmeric also contains a range of non-curcuminoid constituents increasingly recognized as biologically relevant contributors to anticancer activity, particularly volatile-oil sesquiterpenes and related molecules found in rhizome and oleoresin fractions [65,69,70,71,72]. Frequently highlighted examples include turmerones (e.g., α-turmerone and ar-turmerone), β-elemene, germacrone, atlantone, furanodiene, bisacurone, cyclocurcumin, curdione, and the polyphenol calebin A [71,72,73,74]. The recurrent inclusion of these compounds in anticancer studies reflects a gradual shift from a curcumin-centered view of turmeric toward a broader, multi-compound pharmacological perspective [65,72]. Of translational relevance, elemene has been described as a turmeric-derived compound approved for cancer treatment in China, indicating that some turmeric-derived molecules have progressed further in clinical development than curcumin itself, although not specifically in GC [73].

In addition to low-molecular-weight phytochemicals, turmeric contains immunologically active high-molecular-weight fractions. Water-soluble polysaccharides, including turmerosaccharides, have been proposed to contribute to immunomodulatory effects that may be relevant to inflammation-associated gastric carcinogenesis and immune dysfunction during chemotherapy [64,69,70,75]. Notably, comparative in vitro studies suggest that whole turmeric preparations may exert stronger growth-inhibitory effects than isolated curcumin when matched for curcumin content, implying that non-curcuminoid constituents and matrix effects may enhance or reshape biological activity [57,76]. This distinction is particularly important when mechanistic findings from purified curcumin are extrapolated to whole-plant preparations in GC.

#### 3.1.2. *Curcuma longa*: Mechanisms of Anticancer/Chemopreventive Activity in GC

A recurrent chemopreventive theme in the GC-focused literature is that curcumin and turmeric-derived preparations may act upstream of malignant transformation by reducing gastric mucosal injury and chronic inflammation, two processes strongly implicated in gastric carcinogenesis [59,64,77,78]. Curcumin has been described as protective in preclinical models of gastric mucosal injury induced by non-steroidal anti-inflammatory drugs, stress-related ulcerogenic stimuli, and *Helicobacter pylori* infection, thereby supporting its relevance to inflammation-driven gastric tumorigenesis [64,77]. More broadly, curcumin is consistently characterized as a pleiotropic anti-inflammatory and antioxidant molecule capable of acting as both a blocking and a suppressing chemopreventive agent across stages of carcinogenesis [65,78]. At the same time, translational caution is warranted, since nutrition- and pharmacology-oriented studies repeatedly note that experimentally effective exposures may exceed those achievable through diet alone, highlighting a persistent dose-exposure gap [66].

In established GC models, curcumin has been reported to exert direct anti-tumor effects by multitarget inhibition of proliferative, inflammatory, invasive, angiogenic, and survival pathways, with particular emphasis on NF-κB-centered signaling networks [64,78,79]. In GC cell lines such as AGS and SGC-7901, curcumin has been associated with suppression of NF-κB activity, an effect relevant not only to growth inhibition but also to chemosensitization [64,79]. In support of this, Cacciola et al., who specifically addressed combination strategies in GC, proposed that curcumin enhances the therapeutic efficacy of 5-fluorouracil (5-FU) while potentially reducing toxicity, at least in part through downregulation of COX-2 and NF-κB signaling. The same study also suggested that curcumin interferes with the formation of cancer-associated fibroblasts (CAFs), thereby linking its anti-inflammatory effects to a stromal mechanism that may be relevant to chemoresistance in GC [79].

Beyond NF-κB, curcumin has also been linked to other GC-relevant signaling axes. Experimental evidence indicates that curcumin can increase PTEN expression in gastric cancer cells, with associated antiproliferative and pro-apoptotic effects, thereby implicating the PTEN/AKT pathway [80]. In parallel, GC-focused studies have highlighted the regulation of microRNAs, particularly the miR-21/PTEN/AKT axis, as another plausible mechanism through which curcumin influences tumor growth, invasion, and survival [64]. Hedgehog signaling has likewise been discussed in this context, as aberrant Sonic hedgehog/Gli activity is considered relevant to gastric adenocarcinoma biology, and curcumin has been positioned as a compound capable of modulating this pathway in cancer models [64,80]. These observations are consistent with the broader literature describing curcumin as a multi-pathway modulator affecting apoptosis, cell-cycle control, and invasion/metastasis-associated signaling. However, the dominant mechanism appears to be model-dependent [65,78,81].

Importantly, the GC relevance of turmeric extends beyond curcumin. Calebin A, a non-curcuminoid turmeric polyphenol, has attracted particular interest because it has been explicitly linked to drug-resistance phenotypes in GC models [72,82]. In vincristine-resistant gastric cancer cells, calebin A has been reported to inhibit proliferation, induce apoptosis, and promote S/G2/M-phase arrest, while also enhancing vincristine cytotoxicity, suggesting a resistance-modifying phenotype in vitro [72,82]. This observation aligns with broader mechanistic interpretations that frame turmeric-derived polyphenols as regulators of NF-κB signaling and apoptosis-related proteins, providing a rationale for their evaluation in multidrug-resistant settings [59,72,82]. Likewise, volatile non-curcuminoid constituents such as turmerones and elemene are repeatedly recognized as biologically active compounds. They are frequently discussed in the context of formulation strategies to improve solubility and delivery [65,72,77].

Pharmacokinetic limitations remain central to the interpretation of these mechanistic findings. Multiple studies emphasize that curcumin has poor bioavailability, widely regarded as a major reason robust in vitro effects do not readily translate into meaningful in vivo or clinical efficacy without optimized delivery systems [59,66,68,77,78]. Consequently, nanoformulations and other delivery approaches are increasingly considered integral to the translational development of turmeric-derived interventions rather than secondary technical refinements [59,66,68,77]. At the same time, comparisons between purified curcumin and whole turmeric preparations suggest that non-curcuminoid companion molecules may augment or modify bioactivity, reinforcing the importance of clearly distinguishing among purified curcumin, curcuminoid mixtures, and crude turmeric extracts or oleoresins when interpreting GC data [57,72,76].

#### 3.1.3. *Curcuma longa*: Overall Assessment

Overall, recent preclinical evidence supports *Curcuma longa* as a multi-component medicinal plant with plausible GC-relevant chemopreventive and anti-tumor activity. Its proposed effects converge on inflammation control, mucosal protection, and suppression of malignant phenotypes through multitarget modulation of signaling pathways involved in cell survival, apoptosis, cell-cycle progression, and stromal interactions, with NF-κB/COX-2-related signaling representing one of the most consistently discussed mechanistic axes [64,78,79]. Importantly, the biological rationale for turmeric in GC extends beyond curcumin alone. Calebin A has shown activity in vincristine-resistant gastric cancer cells and may therefore be relevant to resistance-oriented research. At the same time, volatile-oil constituents and other non-curcuminoids provide additional mechanistic and formulation-related opportunities [72,73,82].

From a safety perspective, turmeric and curcumin are generally regarded as well tolerated in humans, and curcuminoids are often described in the literature as having a favorable safety profile even at gram-level daily intake [66,67,77]. However, toxicology-oriented animal studies indicate that very high-dose exposure may produce adverse effects, including changes in organ weights and histological alterations, underscoring that “generally safe” should not be interpreted as equivalent to unrestricted dosing [57,63].

Looking ahead, three priorities emerge consistently from the recent literature: first, turmeric-based interventions should be treated as chemically defined, standardized preparations rather than used interchangeably with purified curcumin; second, further GC research should extend beyond curcumin to include non-curcuminoid constituents such as calebin A, particularly in the context of resistance biology; and third, bioavailability-enhancing formulations and clinically realistic exposure assessments should be prioritized, since poor systemic availability remains a major bottleneck between preclinical promise and clinical applicability [59,64,66,68,72,77]. These priorities are especially relevant in GC, where inflammation- and stroma-related mechanisms, including CAF-associated resistance, are increasingly invoked in the curcumin literature but remain insufficiently validated in clinically representative models [68,79].

Overall, curcumin remains one of the better-supported phytocompounds with chemotherapy-modifying activity relevant to gastric cancer. However, much of the available evidence is more consistent with chemosensitization than with rigorously demonstrated formal pharmacological synergy.

### 3.2. Allium sativum (Garlic)

#### 3.2.1. Relevant Phytocompounds of *Allium sativum*

Across recent evidence syntheses on plant-derived interventions in gastric cancer (GC), *Allium sativum* is consistently identified as one of the most extensively investigated edible and medicinal plants, largely because different garlic-derived preparations, including fresh garlic products, oils, and aged or fermented extracts, contain chemically distinct yet biologically active organosulfur compound (OSC) profiles that have been repeatedly evaluated in GC-relevant models [83,84,85]. The principal chemical rationale for garlic’s activity in GC centers on OSCs, although phenolics, flavonoids, saponins, and polysaccharides may also contribute to redox regulation and inflammation-related effects relevant to chemoprevention and tumor control [86,87,88].

From a phytochemical and processing perspective, the bulb (clove) is the primary medicinally relevant part of the plant. In intact garlic tissue, sulfur precursors such as S-allyl-L-cysteine sulfoxide (alliin) and γ-glutamyl-S-allyl-L-cysteine derivatives are spatially separated from the enzyme alliinase. Upon crushing or cutting, alliinase converts alliin into the highly reactive thiosulfinate allicin [86,89,90]. Allicin is a key candidate in the GC literature. However, it is chemically unstable and rapidly decomposes into multiple downstream OSCs, including lipid-soluble allyl sulfides and polysulfides such as diallyl sulfide (DAS), diallyl disulfide (DADS), and diallyl trisulfide (DATS), as well as ajoenes and vinyldithiins, which may predominate in specific processed preparations [86,90,91]. Consequently, different garlic preparations can yield markedly different bioactive exposures. Steam-distilled garlic oil is typically enriched in volatile allyl sulfides; dried garlic powder often contains alliin and DADS; macerates are richer in ajoenes and dithiins; whereas aged garlic extract (AGE) is characterized by comparatively stable, water-soluble OSCs such as S-allyl-L-cysteine (SAC) and S-allylmercaptocysteine (SAMC) [83,86,87,90]. Fermented preparations such as black garlic and aged black garlic extracts are also reported to contain relatively high levels of SAC, SAMC, and antioxidant constituents, reflecting fermentation-driven compositional changes that may be relevant to tolerability and formulation strategies [92,93,94,95,96,97,98,99,100,101,102,103,104,105,106,107].

Although most GC research has focused on bulb-derived preparations, recent phytochemical studies have identified additional cyclic OSCs in garlic leaves, including foliogarlic disulfanes and trisulfanes. These findings suggest that non-bulb plant parts may further expand the OSC chemical space and merit future exploration in gastrointestinal oncology [73,107].

#### 3.2.2. *Allium sativum*: Mechanisms of Anticancer/Chemopreventive Activity in GC

Preclinical evidence most consistently supports garlic-derived OSCs as modulators of proliferation, apoptosis, and cell-cycle progression in established GC models, with allicin and allyl polysulfides being the most frequently discussed compounds [84,85,86,96]. In vitro, allicin has been reported to promote apoptosis in human GC cells, including MGC803 cells, with associated increases in cleaved caspase-3 and activation of stress-related signaling pathways such as p38 MAPK, consistent with an apoptosis-oriented rather than purely cytostatic mechanism [84,85]. Broader hallmark-based syntheses further associate allicin exposure in GC models with G2/M arrest, modulation of endoplasmic reticulum stress, and mitochondria-mediated apoptosis, indicating that allicin activates several coordinated death pathways rather than a single linear mechanism [84,96]. However, most of these findings derive from conventional two-dimensional cell line systems such as AGS, HGC27, and MGC803, and broader garlic studies repeatedly emphasize that the dominant mechanism may vary with preparation type, cell model, and experimental design [83,85,86,89].

Among garlic OSCs, DADS is one of the best-characterized compounds in mechanistic GC studies addressing pathway-level and non-coding RNA-mediated regulation. In GC cell lines and tumor-bearing mouse models, DADS has been linked to anti-proliferative and anti-invasive effects, including increased expression of miR-200b and miR-22, suppression of Wnt1 signaling, and upregulation of miR-34a, as well as reduced PI3K/Akt phosphorylation [97,98]. These observations suggest that DADS may simultaneously affect stemness-, epithelial–mesenchymal transition-, and survival-related programs. In addition, DADS has been associated with G2/M arrest, Chk1-mediated checkpoint signaling, increased histone H3/H4 acetylation, and induction of p21WAF1 in GC cells, as well as dose-dependent antitumor activity in xenograft models [97,98]. Although these findings support the biological plausibility of DADS as a network-active modulator in GC, they also illustrate a recurrent translational problem: similar phenotypic outcomes, such as apoptosis, cell-cycle arrest, and reduced invasion, may arise through different upstream mechanisms depending on the experimental context [83,89,98].

Anti-migratory and anti-invasive effects have also been repeatedly attributed to DATS and related allyl polysulfides. In GC models, DATS has been reported to alter the expression of metastasis-related proteins, including MMP9 and E-cadherin, and to be associated with reduced migration and invasion in SGC-7901-derived xenograft systems [99]. Related studies also describe DATS-induced apoptosis in vivo, suggesting that suppression of invasive behavior may occur alongside direct tumor cell killing rather than as an isolated motility effect [99,100]. These findings are consistent with broader OSC studies describing allyl polysulfides as compounds capable of coupling redox perturbation and stress signaling to both the induction of apoptosis and the attenuation of metastasis-associated traits. However, the balance between pro-oxidant and antioxidant effects remains preparation- and context-dependent [86,87,89].

Water-soluble OSCs derived from AGE and fermented garlic preparations provide an additional mechanistic dimension. AGE and black garlic are repeatedly distinguished from fresh garlic because they are enriched in relatively stable, water-soluble molecules such as SAC and SAMC, which may differ substantially from allicin-rich preparations in both pharmacokinetic behavior and biological effects [86,87,92]. In GC-relevant summaries, SAC and SAMC have been described as suppressing proliferation of gastric adenocarcinoma cells and inducing S-phase-associated cell-cycle arrest, indicating that AGE-like matrices may retain direct anti-proliferative activity even in the absence of allicin as the dominant administered species [99,100]. Studies on black garlic further report inhibition of SGC-7901 growth in vitro and in mouse models, together with increased endogenous antioxidant enzyme activity, including superoxide dismutase and glutathione peroxidase, and induction of apoptosis [92,93]. At the same time, these studies note that biological activity may vary depending on the extraction method and preparation type, reinforcing the conclusion that aged or black garlic should not be treated as a single standardized intervention [83,92].

Garlic also has a plausible chemopreventive role in GC because its biological effects extend beyond direct tumor-cell cytotoxicity. Since *Helicobacter pylori* infection and chronic gastric inflammation are major upstream determinants of gastric carcinogenesis, garlic’s antibacterial, anti-inflammatory, and immunomodulatory properties are frequently discussed in a chemopreventive framework [84,101]. In vitro, allicin-containing ethanolic and acetonic garlic extracts have been reported to inhibit *H. pylori* growth, providing a mechanistic bridge between garlic phytochemistry and risk-reduction hypotheses for infection-associated gastric carcinogenesis [84]. In addition, AGE and related preparations have shown protective effects in preclinical models of chemically induced gastric injury, including reduced microbial burden and improved mucosal recovery, findings often interpreted as supportive evidence for gastric barrier protection and anti-inflammatory activity [92,100]. Garlic extracts and OSCs have also been reported to modulate cytokine secretion and the function of macrophages, lymphocytes, natural killer cells, and dendritic cells. However, several studies caution that the direction of these immune effects may differ across preparations and experimental systems [87,90,102]. This uncertainty is directly relevant for GC translation, since immunomodulation may either support tumor control or generate variability and unintended interactions when garlic-derived products are combined with chemotherapy or immunotherapy [83,89,90].

Allicin has been described in a clinical context as a locally administered agent in progressive gastric carcinoma before gastrectomy, with reported antiproliferative and pro-death effects in resected tumor tissue [86,103,104]. In addition, population-based intervention studies of garlic supplementation have been interpreted as suggesting possible long-term protective associations with GC incidence or mortality in specific contexts. However, such findings remain highly dependent on study design and are generally regarded as provisional [103,104]. In vitro, allicin has also been reported to enhance apoptosis and reverse multidrug-resistance-associated phenotypes in GC cells when combined with 5-fluorouracil, with downregulation of WNT5A, DKK1, MDR1/P-gp, and CD44 [96,105]. While these findings belong more directly to the later discussion on combination strategies, they strengthen the mechanistic plausibility of garlic-derived OSCs as chemosensitizing agents in GC. At the same time, broader garlic pharmacology studies emphasize that interpretation of such results must account for the marked preparation dependence and in vivo instability of allicin, which may substantially affect clinically achievable exposure [86,89,106].

#### 3.2.3. *Allium sativum*: Overall Assessment

Collectively, contemporary GC-focused studies support *Allium sativum* as a high-priority medicinal and functional plant for GC chemoprevention and adjunctive investigation. The strongest preclinical evidence points to OSC-driven suppression of GC cell proliferation, induction of apoptosis, cell-cycle arrest, and attenuation of invasive behavior across commonly used GC cell lines and xenograft models [83,84,85]. Mechanistically, GC studies particularly implicate stress-activated kinases, such as p38 MAPK, in allicin-related apoptosis and survival; epithelial–mesenchymal transition-associated pathways, such as PI3K/Akt and Wnt signaling, in DADS-related effects; and metastasis-associated proteins, such as MMP9 and E-cadherin, in DATS-related responses [85,92,97,99]. AGE- and black-garlic-derived matrices further add a layer of SAC/SAMC-centered biology that may be more compatible with oral standardization and translational development than allicin-centric concepts alone [92,93,99]. A chemopreventive dimension is also supported by in vitro inhibition of *H. pylori* and by mucosal-protective effects in non-neoplastic gastric injury models. However, extrapolating these findings to reduced GC initiation in humans remains inferential rather than definitive [84,100,101].

From a safety and translational perspective, however, the literature also identifies several recurring limitations. First, garlic is highly preparation-dependent: fresh/crushed garlic, oils, powders, macerates, AGE, and black garlic differ substantially in their OSC composition [82]. Second, allicin is chemically unstable, and OSCs are generally highly reactive, which complicates exposure-response mapping and the assignment of a single “active principle” in vivo [86,89,99,106]. Third, clinically relevant herb-drug interactions remain a concern. Research repeatedly reports that garlic supplementation can reduce plasma concentrations of saquinavir in healthy volunteers, underscoring the need for systematic evaluation of interactions with anticancer drugs that share metabolic or transport pathways [84,86,87,89,99]. Additional toxicological considerations, including the rapid disappearance of allicin from the blood and possible oxidative interactions with hemoglobin under certain conditions, further reinforce the conclusion that “natural” should not be equated with automatic benignity at pharmacologically relevant doses [89,106].

Accordingly, future progress in GC-oriented garlic research will require chemically standardized preparations, robust pharmacokinetic/pharmacodynamic bridging, and formulation strategies that stabilize labile OSC chemistry while preserving multi-target biological activity [87,95,107]. In practice, SAC-standardized AGE is often presented as one of the more feasible translational models because of its relative compositional stability [87]. Finally, because the immunomodulatory effects of garlic can vary substantially across preparations and model systems, future GC studies should integrate immune phenotyping with tumor-response endpoints in clinically relevant platforms and include formal interaction testing with standard chemotherapeutics to distinguish true chemosensitization from preparation- or model-dependent artifacts [83,90,105].

Overall, garlic-derived preparations remain promising adjunct candidates in gastric cancer. However, direct evidence of GC-specific chemotherapy interactions remains more limited and less mechanistically well developed than that currently available for curcumin, wogonin, or baicalein.

### 3.3. Glycyrrhiza glabra (Licorice)

Licorice used in traditional medicine is obtained mainly from the dried roots and rhizomes of *Glycyrrhiza glabra* L. [108,109,110,111]. Chemical and pharmacological studies indicate that licorice is a complex phytochemical matrix containing triterpenoid saponins, flavonoids and chalcones, polysaccharides, and other phenolic constituents [108,110,112,113,114]. In the context of gastric cancer, recent research has positioned licorice both as a source of direct anti-GC bioactives and as a potential adjunctive botanical whose constituents may influence chemotherapy responsiveness and tolerability. However, important translational limitations remain, including variability in extracts, compound-specific effects, and limited high-quality clinical data [108,109,110,115].

#### 3.3.1. Relevant Phytocompounds of *Glycyrrhiza glabra*

The licorice constituents most consistently linked to GC-relevant activity belong to two major phytochemical groups: triterpenoid saponins/terpenoids and flavonoids, including chalcones, concentrated mainly in the roots and rhizomes [108,112,113,114]. Among the triterpenoids, glycyrrhizin (glycyrrhizic acid) is widely recognized as a principal marker compound of licorice, whereas 18β-glycyrrhetinic acid (18β-GA) is regarded as a key bioactive aglycone generated through glycyrrhizin biotransformation in humans [108,116]. This distinction is translationally important because preclinical studies have tested both glycyrrhizinic acid and glycyrrhetinic acid, and these related molecules may differ in potency and target profile despite their metabolic connection [108,116].

Within the flavonoid/chalcone fraction, licochalcone A is one of the best-characterized licorice compounds in GC models and has been studied in several human GC cell lines, including AGS, MKN-28, MKN-45, and MGC-803 [108,113,117]. Experimental studies also identify isoliquiritigenin as a licorice-derived chalcone with broad anticancer activity and specific relevance to GC chemosensitization models involving 5-fluorouracil (5-FU) [108,118]. Additional licorice flavonoids, including liquiritin and related scaffolds such as liquiritigenin, have been associated with modulation of apoptosis and autophagy in GC systems, including drug-resistant settings [108,109,117]. At the same time, phytochemical studies emphasize that licorice activity cannot be reduced to a few isolated compounds, since differences in species, extraction method, and fractionation can substantially alter the dominant chemical profile and, consequently, the observed biological effects [108,110,114,119].

#### 3.3.2. *Glycyrrhiza glabra*: Mechanisms of Anticancer/Chemopreventive Activity in GC

Studies support the use of licorice-derived compounds as inhibitors of GC cell proliferation, disrupting the cell cycle and suppressing survival signaling [108,112,113,117]. Licochalcone A has been reported to reduce clonogenic growth and downregulate major G1/S regulators such as cyclin D1 and CDK4, consistent with a proximal anti-proliferative effect [108]. Glycyrrhizinic acid has also shown antiproliferative activity in multiple GC cell lines, including MGC-803, BGC-823, and SGC-7901, indicating that triterpenoid saponins contribute to direct anti-GC activity in addition to the flavonoid/chalcone fraction [108,112]. In mechanistic studies, these effects frequently converge on the PI3K/AKT axis and related cell-cycle and survival pathways [108,113,117].

Apoptosis induction represents another recurrent mechanism. Experimental studies have reported that licochalcone A promotes reactive oxygen species (ROS) generation and apoptotic cell death in GC cells, while modulating the PI3K/AKT/mTOR and MAPK signaling pathways [108,117]. In addition, licochalcone A has been linked to the suppression of glycolytic metabolism through inhibition of hexokinase 2 (HK2) and AKT signaling, thereby linking metabolic disruption to apoptotic vulnerability in GC models [109,117]. Glycyrrhizinic acid has likewise been associated with apoptosis and cell-cycle arrest via modulation of PI3K/AKT signaling, suggesting that chemically distinct licorice constituents may converge on common survival-signaling nodes [108,120]. Overall, the available research supports a multi-target mode of action, although the relative contribution of individual pathways appears to be model dependent [108,113,117].

Licorice-derived compounds have also shown anti-migratory and anti-invasive activity in GC models. Licochalcone A and isoliquiritigenin have both been associated with suppression of metastatic phenotypes and epithelial–mesenchymal transition-related proteins in human GC cells [108,117]. In parallel, 18β-glycyrrhetinic acid has been reported to inhibit invasive and metastatic properties of SGC-7901 cells through a ROS/PKC-α/ERK-related mechanism, indicating that licorice terpenoids may also regulate invasion-associated signaling beyond classical PI3K/AKT survival pathways [108,114]. Although most of these findings derive from in vitro migration and invasion assays, they are consistent with the broader experimental pattern showing that licorice compounds exert combined anti-proliferative, pro-apoptotic, and anti-metastatic effects [114,119].

A major reason licorice is of interest in GC adjunctive research is its reported chemosensitizing potential. Experimental studies indicate that licochalcone A can enhance the antiproliferative effect of 5-FU in GC cells by promoting cell-cycle arrest and apoptosis [108,118]. Isoliquiritigenin has also been reported to increase 5-FU sensitivity in MKN45 GC cells and corresponding xenograft models [118]. In addition, liquiritin has been described as enhancing apoptosis and autophagy when combined with cisplatin, including in drug-resistant GC settings, and as augmenting TRAIL-mediated apoptosis through caspase activation in vitro and in xenograft models [109,117]. Glycyrrhizin has likewise been linked to increased sensitivity to radiation and cisplatin through the regulation of HMGB1, and some combination studies have associated licorice-derived interventions with downregulation of multidrug resistance proteins such as MRP2, MRP3, and MRP5 [108,112]. Together, these findings support the hypothesis that defined licorice bioactives may function as resistance-modifying or apoptosis-restoring agents in GC.

Beyond direct anti-tumor effects, licorice also has plausible chemopreventive relevance through gastric mucosal protection and control of inflammation-associated injury. Since chronic gastric inflammation and *Helicobacter pylori*-associated pathology contribute to gastric carcinogenesis, the anti-inflammatory and mucosal-protective properties of licorice may be biologically relevant upstream of overt malignancy [111]. In an aspirin-induced gastric ulcer model, hot-water licorice extract reduced ulcer burden and mucosal damage, with proposed mechanisms including increased prostaglandin E2 production and enhanced bicarbonate and mucus secretion [111,121]. In supportive-care settings outside GC-specific treatment trials, aqueous licorice extracts have also been reported to reduce the severity of radiotherapy-associated oral mucositis and may improve quality of life. However, stronger clinical evidence is still needed [109,110]. These data support a dual role for licorice in GC-oriented research: direct anti-tumor activity driven by defined phytocompounds, and indirect supportive or chemopreventive potential through mucosal and inflammatory modulation [108,109,110,115].

#### 3.3.3. *Glycyrrhiza glabra*: Overall Assessment

Collectively, current preclinical research supports *G. glabra* as a multi-component medicinal plant with plausible anti-GC activity derived mainly from root/rhizome triterpenoid saponins and flavonoid/chalcone constituents [108,113,114,117,118]. Glycyrrhizin/glycyrrhetinic acid, licochalcone A, isoliquiritigenin, and liquiritin-related compounds have all been associated with GC-relevant effects, including growth inhibition, cell-cycle arrest, apoptosis and autophagy induction, metabolic suppression, and inhibition of invasion or EMT-associated phenotypes, with repeated convergence on PI3K/AKT/mTOR and MAPK signaling networks [108,113,114,117,118,120]. The same body of evidence also supports a chemosensitizing role for licorice constituents, particularly in relation to 5-FU-, cisplatin-, TRAIL-, and HMGB1-associated response pathways [108,109,112,117,118].

From a translational perspective, however, several limitations remain. Licorice in experimental and clinical settings encompasses different *Glycyrrhiza* species and multiple extraction or fractionation approaches, which can substantially alter chemical composition and biological outcome [109,110,119]. In addition, although licorice has been discussed as potentially beneficial for supportive care and treatment tolerability, this promise remains constrained by limited high-quality clinical evidence and the possibility of herb–drug interactions, including modulation of drug-metabolizing enzymes such as the cytochrome P450 system [109,110,115]. Future GC-oriented research should therefore prioritize chemically defined preparations, distinguish clearly between glycyrrhizin- and glycyrrhetinic-acid-dominant exposures, validate synergy and resistance-reversal effects in clinically relevant models, and integrate pharmacokinetic and interaction-aware study designs. Within these constraints, *G. glabra* remains a credible source of candidate adjunct bioactives for GC, but successful translation will depend on rigorous standardization, model selection, and safety assessment [108,109,110,116,117,118].

Overall, licorice-derived compounds show credible chemotherapy-modifying potential in gastric cancer. Still, some supporting evidence involves broader anticancer mechanisms or non-conventional response settings, so the evidence should be regarded as intermediate rather than among the strongest candidates in this review.

### 3.4. Rhus verniciflua Stokes (Syn. Toxicodendron vernicifluum)

#### 3.4.1. Relevant Phytocompounds of *Rhus verniciflua*

*Rhus verniciflua Stokes*, also known as *Toxicodendron vernicifluum*, is a species of Anacardiaceae widely distributed in East Asia and traditionally used for gastrointestinal disorders and cancer-related conditions [122,123,124,125,126,127,128,129]. However, its medicinal use has long been limited by the presence of allergenic urushiol derivatives, especially in the bark [124,127,128,129]. For this reason, more recent developments have focused on detoxified or allergen-reduced preparations, including heat-processed and fermented extracts, while recognizing that processing can substantially alter the phytochemical composition and, therefore, biological activity [124,127,129,130].

Phytochemical studies indicate that the biological activity of *Rhus verniciflua* is mainly associated with a polyphenol- and flavonoid-rich profile, including phenolic acids such as gallic, protocatechuic, caffeic, chlorogenic, p-coumaric, and p-hydroxybenzoic acids, as well as flavonoids and related compounds such as fustin, fisetin, quercetin, kaempferol, taxifolin, eriodictyol, liquiritigenin, butin, and the chalcone butein [123,124,125,128,130,131]. In gastric-cancer-oriented research, bark-derived extracts, particularly ethyl acetate fractions, have often been emphasized because they are enriched in phenolics and flavonoids with measurable bioactivity [125,128]. At the same time, comparative studies across plant parts indicate that urushiol burden is highest in bark. In contrast, lignum, or heartwood, contains abundant phenolic constituents and much lower urushiol content, making these tissues potentially safer sources for anticancer-oriented development [124,127,129].

Processing also appears to be a critical determinant of composition. Experimental comparisons of allergen-free *Rhus verniciflua* preparations produced by heating or fermentation have shown substantial differences in major phenolic markers, including gallic acid, fustin, fisetin, and quercetin, suggesting that the reproducibility of anticancer effects depends strongly on detoxification and manufacturing conditions [124]. Fermentation studies likewise indicate that microbial processing may not only reduce allergenicity but also reshape bioactivity by altering the phenolic profile and pathway-level biological responses [130,132].

#### 3.4.2. *Rhus verniciflua*: Mechanisms of Anticancer/Chemopreventive Activity in GC

The most direct GC-relevant evidence currently available comes from experimental studies using stomach carcinoma cell models treated with ethanol extracts of *Rhus verniciflua*. In these systems, *Rhus verniciflua* extracts have been reported to suppress G1 cell-cycle progression by upregulating p27Kip1 and to induce intrinsic apoptosis, characterized by increased Bax, reduced Bcl-2, cytochrome c release, and activation of caspase-9 and caspase-3 [123]. These effects were accompanied by inhibition of PI3K-Akt/PKB signaling, suggesting that RVS can couple cell-cycle restraint with mitochondrial apoptosis by interfering with a major pro-survival pathway [123]. Although this provides a biologically plausible anti-GC mechanism, the available evidence remains limited by incomplete standardization of extract composition and the predominance of in vitro systems [123].

Additional mechanisms relevant to chemoprevention emerge from inflammation- and immune-related studies. In THP-1 monocytic cells, RVS extracts have been shown to reduce the expression of inflammatory mediators, including metalloproteinases, inducible nitric oxide synthase, and COX-2 through inhibition of NF-κB and MAPK phosphorylation [133]. This is mechanistically relevant because chronic inflammation is a well-established contributor to gastric carcinogenesis, and NF-κB is a central regulator of cytokine signaling, tissue remodeling, and inflammatory persistence [133,134].

A distinct, translationally relevant mechanism has emerged from immune checkpoint screening studies. *Rhus verniciflua* bark extract has been reported to inhibit PD-1/PD-L1 and CTLA-4/CD80 interactions in competitive ELISA assays, with the ethyl acetate fraction showing the strongest activity [125]. Several isolated constituents, including eriodictyol, fisetin, quercetin, and liquiritigenin, contributed to PD-1/PD-L1 blockade, whereas protocatechuic acid, caffeic acid, taxifolin, and butin contributed to CTLA-4/CD80 inhibition [125]. These findings do not demonstrate functional antitumor immunity in gastric tumor systems. Still, they suggest that RVS-derived phenolics may influence immune-regulatory pathways that are clinically relevant in modern GC treatment [125].

Butein is recognized as a *Rhus verniciflua*-derived chalcone with broad anticancer potential and links to JAK/STAT-oriented anticancer strategies [131]. Likewise, fustin is one of the major flavonoid markers of *Rhus verniciflua* and is abundant in heartwood, and is directly cited in experimental pharmacology, which relates mainly to gastric ulcer protection rather than gastric cancer [124,129]. Thus, the most defensible conclusion at present is that *Rhus verniciflua* has extract-level anti-GC activity, while the exact constituents primarily responsible for the observed gastric cancer phenotypes remain insufficiently resolved [123,124,125,128,131].

#### 3.4.3. *Rhus verniciflua*: Overall Assessment

Overall, current experimental evidence supports *Rhus verniciflua* as a polyphenol- and flavonoid-rich medicinal species with measurable preclinical activity in gastric carcinoma models [123,124,125,128]. The most direct anti-GC effects reported to date include G1 arrest associated with p27Kip1 upregulation, mitochondrial apoptosis, and suppression of PI3K-Akt/PKB signaling in gastric carcinoma cells [123]. Additional studies suggest that *Rhus verniciflua* also influences inflammatory and immune-regulatory processes relevant to gastric carcinogenesis, including NF-κB/MAPK-mediated inflammatory signaling and in vitro checkpoint-ligand interactions involving PD-1/PD-L1 and CTLA-4/CD80 [125,133,134,135].

From a translational perspective, the major limitation remains safety-related standardization. Urushiol-associated allergenicity restricts the use of crude *Rhus verniciflua* preparations, particularly from bark, and makes detoxification or the use of lower-urushiol tissues such as heartwood especially important [124,127,129]. However, detoxification is not a neutral manufacturing step: different allergen-removal approaches can substantially alter levels of key phenolic compounds, thereby modifying bioactivity [124]. Supportive toxicology data are available for some urushiol-free fermented preparations administered orally at high, repeated doses in rodents. Still, these data are indirect and cannot yet be generalized to all *Rhus verniciflua* products intended for oncology [136].

Accordingly, future GC-oriented *Rhus verniciflua* research should prioritize chemically standardized, urushiol-quantified preparations; link marker compounds to biological activity through bioactivity-guided fractionation; and validate proposed mechanisms in more disease-relevant systems, including in vivo gastric tumor models and immune-competent platforms if checkpoint-related effects are pursued [124,125,137]. Within these constraints, *Rhus verniciflua* remains a plausible source of candidate adjunct bioactives for GC, but successful translation will depend on careful control of extract composition, safety, and mechanistic reproducibility [123,124,137].

At present, *Rhus verniciflua* is better supported as a source of general anticancer and inflammation- or immune-related activity than as a well-substantiated candidate for gastric-cancer-specific chemotherapy synergy.

### 3.5. Camellia sinensis (Green/Black/Oolong Tea)

#### 3.5.1. Relevant Phytocompounds of *Camellia sinensis*

*Camellia sinensis* is the botanical source of green, black, and oolong tea, which differ primarily by processing and oxidation rather than by plant origin [138,139]. From an anticancer perspective, the most relevant constituents are tea polyphenols, particularly flavan-3-ols (catechins), although the relative abundance of individual compounds varies substantially across tea types and preparation methods [139,140]. Green tea is especially rich in catechins, with (−)-epigallocatechin-3-gallate (EGCG) generally regarded as the dominant and most intensively studied constituent, alongside epigallocatechin (EGC), epicatechin-3-gallate (ECG), and epicatechin (EC) [140,141,142,143]. However, experimental research increasingly indicates that green tea extract (GTE) should be viewed as a multicomponent system rather than as an EGCG-equivalent intervention, because the biological activity of whole extracts may depend on the compositional balance among multiple phytochemicals [140,141].

In green tea leaves, polyphenols may account for a substantial fraction of dry weight, which partly explains why extraction method, solvent, purification, and formulation strongly affect the concentrations delivered in preclinical studies [142,143,144]. Phytochemical profiling studies have also shown that cultivars differ in catechin yield and that non-aqueous extraction methods may recover a broader range of constituents than simple aqueous preparations [144,145]. In addition to catechins, tea extracts contain flavonols, proanthocyanidins, and phenolic acids, further widening the biologically plausible target space beyond EGCG-centered mechanisms [140,145].

By contrast, black tea is characterized by oxidation-derived polyphenols, especially theaflavins and thearubigins, which arise during fermentation and may engage biological pathways that overlap but are not identical to those of catechin-rich green tea [139,140]. Oolong tea generally occupies an intermediate position, reflecting partial oxidation. This distinction is important for translational interpretation because “tea” is often discussed in generic terms. In contrast, green-, black-, and oolong tea interventions may represent distinct chemical exposures with different pharmacological consequences [139,140].

Tea preparations also contain non-polyphenolic constituents that may influence both efficacy and safety. These include methylxanthines such as caffeine, amino acids such as L-theanine and GABA, pigments, polysaccharides, and saponins [138,145]. Because these compounds frequently co-occur with polyphenols unless explicitly removed, comparisons across “polyphenol-rich” tea preparations can be misleading without detailed chemical characterization [138,141,145]. Overall, current evidence supports treating *C. sinensis* interventions as a spectrum ranging from beverage-like infusions to catechin-enriched extracts and purified EGCG, rather than as a single exposure class [138,141,142].

#### 3.5.2. *Camellia sinensis*: Mechanisms of Anticancer/Chemopreventive Activity in GC

Experimental studies consistently identify tea catechins, particularly EGCG, as biologically plausible chemopreventive and therapeutic candidates for gastric cancer, although the strength of evidence varies across mechanistic, preclinical, and epidemiologic contexts [142,143,146]. Some epidemiologic analyses have suggested a modest reduction in stomach-cancer risk among individuals with high green-tea intake. In contrast, others have found weak or null associations, indicating that human data remain inconsistent and that mechanistic plausibility should not be equated with proven preventive benefit [142]. Accordingly, tea bioactives are best positioned as multi-target candidates with supportive preclinical evidence but still uncertain translational effect size in GC [142,143,146].

One of the most frequently discussed properties of tea polyphenols is their ability to modulate redox balance and inflammation-related signaling. EGCG has been widely described as possessing antioxidant and anti-inflammatory activities and as influencing gene expression programs relevant to carcinogenesis [143,147]. This is potentially relevant to GC, where chronic inflammation and oxidative stress contribute to tumor initiation and progression. However, the redox biology of nutraceutical antioxidants is context-dependent. Experimental work suggests that tea polyphenols may sensitize tumor cells under certain conditions but may also interfere with treatment response if their antioxidant activity unfavorably alters cytotoxic redox stress [148,149].

Tea polyphenols have also been repeatedly linked to core anti-tumor phenotypes, including induction of apoptosis, modulation of autophagy, and cell-cycle arrest. Experimental studies in cancer models describe EGCG as capable of activating caspases, suppressing proliferative signaling, and modulating NF-κB-associated pathways relevant to both tumor survival and inflammatory persistence [141,150,151]. Whole green tea extract has likewise been reported to induce apoptosis in malignant cells, suggesting that extract-level activity may reflect cooperation among multiple constituents rather than a single-catechin mechanism [141]. These processes are directly relevant to GC because dysregulated apoptosis competence, persistent NF-κB activation, and altered cell-cycle control are central features of gastric tumor biology and may also influence chemosensitivity [148,149,150].

Another major mechanistic theme involves modulation of growth factor receptors, kinase networks, angiogenesis, and metastasis-associated signaling. EGCG has been reported to influence membrane-associated targets such as EGFR, as well as downstream pathways involving ERK1/2, p38 MAPK, NF-κB, and VEGF, thereby linking tea catechins to the regulation of proliferation, angiogenesis, and tumor progression [150,151]. Additional studies have shown that EGCG can inhibit matrix metalloproteinases, including MMP-2, MMP-3, and MMP-9, through both direct interaction and transcriptional repression, providing a plausible mechanism for reduced invasion and metastatic behavior [142,151]. These findings support a coherent rationale for including tea polyphenols in GC-oriented chemoprevention and adjunct-therapy research, namely that they may exert pleiotropic pressure on inflammatory, proliferative, angiogenic, and invasive pathways simultaneously [142,150,151].

Epigenetic regulation is another attractive, though still translationally challenging, mechanism. Experimental studies have linked EGCG to the inhibition of DNA methyltransferases, the modulation of histone acetylation-related processes, and the broader regulation of gene expression and non-coding RNA networks relevant to carcinogenesis [147,152]. In principle, these properties make tea catechins attractive candidates for long-term chemopreventive intervention, particularly in settings with reversible epigenetic dysregulation. In practice, however, the same issue that affects many nutraceuticals limits these hypotheses: poor and variable bioavailability [147,151]. Consequently, mechanistic claims based on direct in vitro exposure may not be testable in humans without optimized formulations and pharmacokinetic/pharmacodynamic anchoring [147,151]. This has driven interest in nanodelivery systems, improved extract formulations, and rational co-administration strategies to increase systemic exposure and target engagement [9,142,147].

A persistent methodological problem in this field is the tendency to use “tea,” “green tea,” “green tea extract,” “catechins,” and “EGCG” almost interchangeably, despite their substantial chemical differences. Recent phytochemical and metabolomics studies suggest that this simplification can undermine reproducibility because extract composition, constituent stability, and exposure kinetics may strongly influence both potency and mechanism [141,145,151]. For GC research, mechanistic and translational claims are most credible when the intervention chemistry is clearly defined, and the preclinical study design explicitly addresses dose realism, formulation, and chemotherapy interaction endpoints [141,142,148,149,151].

#### 3.5.3. *Camellia sinensis*: Overall Assessment

Taken together, current experimental evidence supports *Camellia sinensis* as a high-priority medicinal and dietary plant for GC-oriented chemoprevention and adjunct-therapy research, as it provides a chemically diverse polyphenol mixture with repeatedly reported anticancer-relevant activities [139,140,141,142,143,147,150,151,152]. Catechin-rich green tea preparations, especially those containing EGCG, are most strongly linked to apoptosis induction, growth arrest, modulation of NF-κB- and MAPK-related signaling, regulation of EGFR/VEGF-associated pathways, inhibition of MMP-dependent invasion, and possible epigenetic reprogramming [142,147,150,151,152]. Black- and oolong-tea chemotypes are also relevant, but should not be treated as pharmacologically interchangeable with green tea because processing substantially reshapes the polyphenol profile [139,140].

At the same time, the translational limitations are substantial. Epidemiologic associations between tea consumption and stomach cancer risk remain inconsistent, and the mechanistic plausibility of EGCG-centered mechanisms is constrained by its instability and limited bioavailability [142,147,151]. Safety interpretation also requires nuance: tea as a beverage and high-concentration green tea extracts are not equivalent exposures. Although tea-derived preparations are often regarded as relatively well-tolerated, concentrated oral green tea extracts have been associated with hepatic adverse reactions in clinical settings, particularly when delivered as supplements with variable catechin composition or in multi-ingredient products [138]. This distinction is especially important in GC patients, who may already receive hepatotoxic chemotherapy or multiple concomitant medications [138,148,149].

For future GC-oriented research, three priorities appear particularly important. First, rigorous phytochemical characterization and extract standardization are essential because compositional balance may materially influence biological response [138,141,145]. Second, formulation and PK/PD-guided strategies to overcome low bioavailability should be integrated early rather than added after efficacy claims are made [9,142,147]. Third, because tea polyphenols may either potentiate or interfere with chemotherapy depending on context and concentration, future studies should explicitly evaluate interaction directionality, define exposure–response relationships, and assess drug–nutrient interaction liabilities with the same rigor expected for drug-development pipelines [148,149,153]. Under these conditions, *C. sinensis* remains a credible source of candidate GC-relevant bioactives, but meaningful translation will depend on standardized chemistry, realistic dosing, and interaction-aware preclinical design [141,148,149,151].

Overall, EGCG provides supportive evidence for gastric-cancer-specific chemosensitization, particularly in 5-fluorouracil-resistant models. Still, the current evidence base remains narrower and less mechanistically developed than that for curcumin or Scutellaria-derived flavones.

### 3.6. Syzygium aromaticum (Clove)

#### 3.6.1. Relevant Phytocompounds of *Syzygium aromaticum*

*Syzygium aromaticum* (clove; Myrtaceae) is widely used as a culinary spice and in traditional medicine. The dried unopened flower buds are the primary medicinal and commercial material, the principal source of essential oil, and a source of polyphenol-rich extracts [154,155]. Phytochemical studies consistently identify clove essential oil (CEO) as dominated by the phenylpropanoid eugenol, which commonly accounts for approximately 70–90% of the oil, together with eugenyl acetate and the sesquiterpene β-caryophyllene; α-humulene has also been described as a relevant terpenoid constituent in some preparations. Although the relative abundance of these compounds varies with plant origin and extraction method, this pattern supports a eugenol-centered pharmacological rationale. It indicates that the CEO is a multicomponent mixture rather than a single-compound intervention [154,155,156,157].

Beyond the volatile oil fraction, clove buds and their derived extracts also contain non-volatile phenolics, including gallic acid and ellagic acid, as well as flavonoids such as quercetin and kaempferol [154,155,158]. This distinction is important because clove-based interventions used in preclinical oncology are chemically heterogeneous, ranging from whole-bud powders and hydroalcoholic extracts to CEO, enriched fractions, and post-distillation residues. Experimental work has shown that even the residue remaining after hydrodistillation retains substantial phenolic content, with gallic acid and ellagic acid identified as major constituents [154,155,159]. Consequently, different clove preparations may expose cells or tissues to markedly different proportions of eugenol versus non-volatile polyphenols, which can substantially alter the observed biological response [154,155,159].

Additional constituents such as oleanolic acid and other minor phenolics or terpenoids have also been reported in clove phytochemical inventories and may contribute to anticancer activity [158,160,161]. However, compared with eugenol and, to a lesser extent, β-caryophyllene or eugenyl acetate, the GC-specific evidence for these minor compounds remains limited and is mostly extrapolated from broader anticancer research rather than from stomach-focused systems [155,158,161].

#### 3.6.2. *Syzygium aromaticum*: Mechanisms of Anticancer/Chemopreventive Activity in GC

The most direct gastric-cancer-focused rationale for clove centers on eugenol. Experimental studies across cancer models, including gastric cancer, have linked eugenol to anti-proliferative activity, cell-cycle perturbation, induction of apoptosis, and suppression of migration, metastasis, and angiogenesis-related phenotypes [155,162,163]. Mechanistically, eugenol has been linked to oxidative-stress-related signaling, DNA-damage-associated responses, mitochondrial dysfunction, and caspase activation, providing a coherent framework for its cytotoxic effects in malignant cells [157,162,164]. These mechanisms are not unique to GC, but they are highly relevant to gastric cancer because they intersect with pathways that also influence tumor survival and response to therapy.

At the same time, a major limitation of the current literature is that detailed pathway and dosing data for clove-derived preparations often come from non-gastric models, especially colorectal cancer. For example, a recent study using an ethanolic extract prepared from the residue remaining after clove hydrodistillation demonstrated selective anti-proliferative and pro-apoptotic effects in p53-wild-type HCT116 colorectal cancer cells, together with increased Annexin V/PI positivity, caspase-3/7 activation, sub-G1 accumulation, p21 upregulation, and cyclin D1 downregulation. Although this is not a GC model, it illustrates two points highly relevant to gastric-cancer translation: first, clove-derived preparations other than CEO may retain biologically meaningful anticancer activity; second, tumor genotype may strongly influence responsiveness, a consideration that is equally important in molecularly heterogeneous GC [159].

Similarly, modulation of the PI3K/Akt/mTOR pathway has been proposed as a major mechanism underlying clove-associated apoptosis and growth suppression. Experimental studies have shown that clove-derived active fractions can induce apoptosis and autophagy while suppressing PI3K/Akt/mTOR signaling in cancer cells. These findings provide mechanistic support for future GC-directed studies [154,158].

Additional information from other tumor models further supports the broader anticancer plausibility of clove while highlighting design-related translational issues. In a carcinogenesis study, dietary administration of clove buds reduced tumor frequency and altered the expression of apoptosis- and proliferation-related proteins, including decreased Bcl-2 and increased Bax and caspase-3. In vitro, clove ethanol extract produced anti-proliferative and pro-apoptotic effects, but the same study also noted practical limitations related to ethanol vehicle toxicity at higher concentrations. These findings are instructive because they show that clove preparations can modulate tumor biology in vivo and that extract dosing and solvent constraints may materially affect the interpretation of preclinical results [165].

Formulation may also influence apparent selectivity. A preliminary nanoemulsion study using clove bud essential oil reported apoptosis-associated changes in HT-29 colon cancer cells, including caspase-3 upregulation and an increased SubG1 fraction, while showing relatively limited toxicity in a normal fibroblast line and evidence of cytoprotective effects in mouse liver. These findings support the translational idea that delivery systems may alter both efficacy and safety of hydrophobic clove-derived mixtures [166,167].

Clove also has chemopreventive and gastroprotective properties that may act upstream of malignant transformation. Experimental studies have described anti-ulcer effects of eugenol and clove-derived preparations, partly attributed to increased gastric mucus production and improved mucosal barrier resistance [155]. An aqueous clove extract has also been reported to ameliorate ethanol-induced gastric mucosal injury in rats, with proposed mechanisms including antioxidant effects, increased prostaglandin E2 production, and reduced inflammatory cell infiltration and epithelial loss [154]. Clove constituents can modulate oxidative stress and inflammatory injury in stomach tissue, which is relevant to chemopreventive framing [154,155].

Finally, antimicrobial activity against *Helicobacter pylori* provides another mechanism relevant to the stomach. A study using clinical isolates of cytotoxin-gene-producing drug-resistant *H. pylori* showed that *S. aromaticum* extracts had greater anti-*H. pylori* activity than comparator herbal extracts, identified eugenol as a major phenolic constituent in the methanolic extract, and reported no IC_50_ in PBMCs up to the highest tested concentration. These results support the feasibility of stomach-relevant antimicrobial testing for clove preparations and underline the importance of paired efficacy–toxicity evaluation [168].

#### 3.6.3. *Syzygium aromaticum*: Overall Assessment

Overall, current experimental research supports Syzygium aromaticum as a multi-constituent medicinal plant with plausible GC-relevant chemopreventive and anticancer potential [154,155,156,157,158,159,162]. CEO is dominated by eugenol, while non-volatile extracts also contain phenolic acids and flavonoids, such as gallic and ellagic acids, indicating that different clove preparations may differ substantially in their biological profiles [154,155,156,157,158,159]. Across preclinical cancer studies, the most consistently reported mechanisms include induction of apoptosis, cell-cycle perturbation, modulation of oxidative stress, and regulation of pro-survival signaling pathways such as PI3K/Akt/mTOR. However, detailed stomach-specific mechanistic datasets remain relatively sparse [154,155,158,159,162,163,164]. Clove also exhibits stomach-relevant gastroprotective and anti-*H. pylori* properties, which strengthen its rationale as a candidate chemopreventive plant in GC-oriented research [154,155,168].

From a translational perspective, several limitations remain. Safety and biological response appear to be highly preparation-dependent, and the commonly cited use of clove as a food should not be taken as evidence that all concentrated clove extracts or eugenol-rich formulations have a wide therapeutic window [164,168,169]. Experimental work suggests that eugenol may exhibit both antioxidant and pro-oxidant behavior depending on dose and cellular context, which is particularly relevant when considering combination strategies with cytotoxic therapy [164]. In addition, the available literature repeatedly highlights the need for better data on the bioavailability, dosing, tissue accumulation, and toxicity of clove products intended for medicinal use [167,169].

Accordingly, future GC-oriented studies should prioritize standardized preparation types, clearly distinguishing CEO from hydroalcoholic extracts and post-distillation phenolic residues; validate efficacy in gastric-cancer-specific preclinical systems; and integrate pharmacology-informed dosing and delivery strategies, including emulsion- or nanoformulation-based approaches where appropriate [154,155,159,166,167]. Such work is necessary to determine whether clove-derived bioactives can be translated credibly into GC prevention or adjunct-treatment settings, rather than remaining supported mainly by cross-cancer mechanistic generalization [162,169].

Eugenol-based systems appear promising, particularly in cisplatin-combination settings. However, the current evidence remains relatively limited and formulation-specific, so this candidate should still be viewed as preliminary compared with the strongest-supported phytochemicals in this review.

### 3.7. Marsdenia tenacissima (Tongguanteng; Source Herb of Xiaoaiping/Tongguanteng Injection)

#### 3.7.1. Relevant Phytocompounds of *Marsdenia tenacissima*

*Marsdenia tenacissima* (Roxb.) Wight & Arn. is used in traditional Chinese medicine for malignant diseases, and modern pharmaceutical preparations derived from this herb, particularly Xiaoaiping and Tongguanteng injections, have been used in China as adjuncts in several cancers, including gastric cancer [170,171,172,173]. A recurring translational issue is that different studies describe different medicinal source materials, most often the stem/caulis but occasionally the root, which has direct implications for composition, standardization, and reproducibility [171,172,173,174,175]. This point is especially relevant because *Tongguanteng* injection has been described as a water extract of the stem. In contrast, *Xiaoaiping* has also been reported as root-derived in some clinical studies, despite other sources classifying it as stem-derived [170,171,172,173,174,175].

The phytochemical class most consistently linked to anticancer activity is the family of C21 steroidal glycosides/saponins, also described as polyoxypregnane glycosides, which are widely regarded as the principal antitumor constituents of *M. tenacissima* extracts and Xiaoaiping-related preparations [176,177,178]. Within this group, tenacigenin-type aglycones, such as tenacigenin A and tenacigenin B, and tenacissoside-type glycosides, including tenacissosides B, G, H, and I, are repeatedly reported in phytochemical and metabolomic studies [179,180,181]. Experimental work further indicates that compound-specific activity is not uniform across this class. Some tenacissosides display marked antitumor activity, whereas others appear considerably less active in vitro, underscoring that “C21 steroidal saponins” should not be treated as a pharmacologically homogeneous category [182].

In addition to C21 steroidal glycosides, *M. tenacissima* extracts contain caffeoylquinic acids and caffeic acid derivatives, including cryptochlorogenic, chlorogenic, and neochlorogenic acids, as well as caffeic acid itself. These compounds have been quantified in tissue distribution studies after oral administration [179]. Analytical studies have also identified triterpenoid and sterol markers, including α-amyrin, betulin, and β-sitosterol, supporting broader phytochemical complexity relevant to quality control and cross-study comparability [183,184]. Processing can further alter the chemical profile. Fermentation-based studies have shown that microbial treatment may increase total saponin content, enrich aglycones, and modify metabolite distribution, which is particularly important given the recognized low oral bioavailability of many *M. tenacissima* constituents [180,181,184]. Thus, as with other medicinal plants, *M. tenacissima* should be viewed not as a single exposure entity but as a family of products whose biological activity depends strongly on plant part, extraction method, and post-processing conditions [171,174,180,181,184].

#### 3.7.2. *Marsdenia tenacissima*: Mechanisms of Anticancer/Chemopreventive Activity in GC

The most direct GC-specific mechanistic evidence concerns C21-enriched fractions derived from *M. tenacissima*. In human GC cell lines, including BGC-823, SGC-7901, and AGS, a refined C21 fraction significantly inhibited proliferation and induced cell-cycle arrest and apoptosis. Experimental data linked these effects to oxidative stress, as evidenced by increased reactive oxygen species generation and altered superoxide dismutase and hydrogen peroxide levels, supporting a redox-dependent apoptotic mechanism. At the protein level, this treatment increased cleaved PARP and BAX while reducing Bcl-2 and phosphorylated AKT, indicating engagement of mitochondrial apoptosis and suppression of AKT-associated survival signaling. Importantly, inhibition of autophagy with chloroquine enhanced oxidative stress and apoptosis in these models, suggesting that autophagy may function as a protective or resistance-associated pathway against *M. tenacissima*-induced cytotoxicity in GC cells [171,174,176,180,181,184,185].

Additional mechanistic themes emerge from broader experimental research and map well onto GC biology, even when not validated directly in gastric models. Anti-angiogenic effects have been repeatedly proposed for M. tenacissima extracts, with studies reporting downregulation of VEGF and MMP-2/-9, molecules centrally involved in tumor vascularization, invasion, and progression [171,185]. Experimental research across cancer models has also linked M. tenacissima to apoptosis-network regulation via caspase activation, BAX/Bcl-2 shifts, and modulation of PI3K/AKT/mTOR signaling, findings consistent with the GC-cell data showing reduced p-AKT and increased pro-apoptotic markers upon C21-fraction exposure [176,178]. Anti-invasive and anti-metastatic effects have likewise been reported, where isolated C21 steroidal saponins promoted ROS-associated mitochondrial dysfunction, cytochrome C release, and downregulation of MMP-2/-9, thereby impairing migration and invasion [186]. Taken together, the strongest experimentally anchored GC mechanism is a C21-driven axis involving oxidative stress, apoptosis, and suppression of survival pathways, whereas anti-angiogenic and anti-invasive effects remain plausible [171,176,178,185,186].

From a translational perspective, *M. tenacissima* has generated substantial clinical interest as an adjunct in GC, although the evidence base remains methodologically limited. Clinical studies in China have reported that oral or injectable *M. tenacissima* preparations, used alongside chemotherapy, may improve response rates, performance status, inflammatory profiles, and certain toxicity outcomes in advanced GC. However, these conclusions are constrained by variable study quality, frequent risk of bias, and incomplete reporting of product-specific safety [171,187]. A multicenter randomized trial assessing Xiaoaiping for chemotherapy-induced thrombocytopenia in non-small cell lung cancer and GC did not demonstrate a clear platelet benefit in the GC subgroup. However, common laboratory safety parameters did not differ significantly between groups during the study period [174]. These findings suggest that *M. tenacissima* may have clinically relevant adjunctive effects, but also highlight the need to interpret current efficacy claims cautiously until better-designed and better-standardized trials become available [171,174,187].

Pharmacokinetic and formulation-related issues add another layer of complexity. After oral administration in rats, quantified tenacissosides and caffeoylquinic/caffeic acids showed rapid tissue distribution, with particularly high stomach exposure reported for both organic acids and steroidal components [179]. This finding supports the plausibility of direct gastric exposure after oral dosing, at least in animal models. However, other studies emphasize that many C21 steroidal constituents have poor oral bioavailability in humans, and that fermentation or other processing methods may increase saponin content and improve antitumor activity [180,181]. This creates an important translational tension: the same herb may produce markedly different local gastric and systemic exposures depending on whether the formulation is oral or injectable, stem- or root-derived, and fermented or non-fermented [171,174,179,180,181]. Such variability needs to be explicitly addressed when interpreting preclinical and clinical findings in GC.

#### 3.7.3. *Marsdenia tenacissima*: Overall Assessment

Overall, the strongest GC-specific preclinical evidence supports C21 steroidal glycoside-enriched fractions from *M. tenacissima* as inhibitors of gastric cancer cell growth through cell-cycle arrest, oxidative-stress-associated apoptosis, and suppression of AKT-related survival signaling. The observation that autophagy inhibition enhances this cytotoxicity further suggests that autophagy may act as a functional resistance buffer in GC cells exposed to *M. tenacissima* constituents [176]. Broader experimental research also supports anti-angiogenic, anti-invasive, and apoptosis-regulating mechanisms involving VEGF, MMP-2/-9, PI3K/AKT/mTOR, and BAX/Bcl-2-associated pathways, which are conceptually consistent with GC progression [171,176,178,185,186].

Clinically, *Xiaoaiping* and related *M. tenacissima* preparations remain of interest because studies in advanced GC suggest possible benefits when combined with chemotherapy, including improved response metrics, performance status, and some inflammatory or toxicity-related outcomes [171,187]. Nevertheless, these conclusions are limited by generally low trial quality, incomplete safety reporting, and likely heterogeneity in botanical source and extract standardization [171,174,187]. Safety interpretation should also remain balanced. Although some clinical data support short-term tolerability, experimental work has shown that crude *M. tenacissima* extract can induce dose-dependent erythrocyte injury phenotypes under certain conditions, raising a plausible concern that systemic exposure may have off-target hematologic effects that are not fully captured in incompletely reported clinical studies [174,182]. In addition, evidence that *M. tenacissima* contains multidrug-resistance-reversing constituents affecting ABC transporter-related pathways further underscores the need for rigorous herb-drug interaction assessment in GC settings [177,188].

Future GC-oriented research should therefore prioritize chemically explicit standardization of preparations, including clear documentation of source material, extraction methods, and marker constituents such as tenacissosides and C21 aglycones; validation in modern GC-relevant models that can assess pathway-conditional effects, such as autophagy dependence; and higher-quality clinical trials with transparent randomization, blinding, and adverse-event reporting [171,176,179,187]. Because herbal products may also be marketed in processed forms that are difficult to authenticate, improved product identification and reproducibility are essential to determine whether the observed benefits of M. tenacissima reflect a consistent drug-like exposure to C21 steroidal glycosides or heterogeneous mixtures that vary across products and studies [183,189].

*Marsdenia tenacissima*-derived preparations remain plausible adjunctive candidates, but the current evidence base remains relatively limited and should be interpreted as supportive rather than definitive.

### 3.8. Scutellaria baicalensis Georgi (Baikal Skullcap; Huang-Qin)

#### 3.8.1. Relevant Phytocompounds of *Scutellaria baicalensis*

The medicinal material most consistently associated with the anticancer and chemopreventive literature on *Scutellaria baicalensis* is the dried root, commonly designated as *Scutellariae radix* or *Radix Scutellariae* (*Huang-Qin*). Available studies identify this root as a flavone-rich phytochemical matrix in which both aglycones and their predominantly glucuronidated derivatives constitute the best-characterized bioactive fraction, a profile widely regarded as central to the plant’s pharmacological activity, including its anticancer effects [190,191]. Across pharmacognostic, pharmacokinetic, and phytochemical studies, the principal flavones most consistently emphasized are baicalein, wogonin, and oroxylin A, together with their corresponding glycosides baicalin, wogonoside, and oroxylin A-7-glucuronide [190,191,192]. These compounds are repeatedly presented as the major polyphenolic determinants linking the chemical profile of Chinese skullcap root to its reported anticancer activity, supporting their prioritization as candidate protective phytocompounds in GC-oriented discussions [192].

A recurrent phytochemical feature of *S. baicalensis* is the enrichment of characteristic 4′-deoxyflavones, particularly baicalein/baicalin and wogonin/wogonoside, in the root tissue [193,194]. This tissue specificity is relevant for reproducibility and extract standardization because aerial parts generally contain substantially lower levels of these signature flavones than the medicinal root [193,194]. Importantly, flavone abundance is not fixed, but can be modified by cultivation conditions. Controlled-environment studies indicate that blue-dominated light can increase root baicalein and wogonin levels while modestly reducing the corresponding glycosides, implying that agronomic variables may alter the relative abundance of candidate protective constituents before extraction even begins [194]. Analytical profiling studies based on modern LC–MS approaches likewise identify baicalin and baicalein as major constituents of skullcap preparations, while also detecting additional flavones such as chrysin, which supports the view that experimental skullcap extracts should generally be interpreted as multi-flavone systems rather than as functional equivalents of a single purified compound [195].

Beyond these major flavones, additional compounds recur in the phytochemical literature and may contribute to biological activity relevant to digestive system tumors. These include scutellarein and scutellarin, which are more often discussed as 4′-hydroxyflavones, as well as other flavonoids highlighted in GC-related network pharmacology analyses, such as acacetin and moslosooflavone [190,193,194,196]. At the same time, the practical availability of individual compounds may differ substantially. Wogonin, for example, may occur at relatively low abundance in the root, with reports indicating that its content often does not exceed approximately 0.5%. In contrast, wogonoside can be present at higher levels and may therefore serve as a practical precursor for hydrolytic preparation of wogonin when gram-scale purified material is required for mechanistic studies [197].

Quality control issues are directly relevant to any claim of biological protection or anticancer activity. Pharmacopoeial and horticultural sources indicate that baicalin has been widely used as a marker constituent for medicinal-grade skullcap, implicitly favoring baicalin-rich root material when comparing products or preclinical extracts across studies [198]. Complementary pharmacological analyses likewise describe baicalin as one of the principal marker flavonoids of *S. baicalensis* root and emphasize that formulation and processing can materially influence pharmacokinetic behavior [199]. Taken together, these findings indicate that the biological interpretation of “skullcap” depends strongly on the plant part used, extraction procedure, and chemical standardization of the final preparation [198,199].

#### 3.8.2. *Scutellaria baicalensis*: Mechanisms of Anticancer/Chemopreventive Activity in GC

Current evidence supports *S. baicalensis* as a multitarget medicinal plant with plausible anticancer and chemopreventive relevance in gastric carcinogenesis. In the most recent GC-proximal syntheses, *S. baicalensis* preparations and major flavones are reported to suppress tumor-cell proliferation, induce programmed cell death pathways, including apoptosis and autophagy, modulate antitumor immune responses, and enhance chemosensitivity in digestive-system tumor models, including GC [196]. Although the underlying preclinical literature is heterogeneous, encompassing different extract types, isolated compounds, purity grades, and experimental conditions, the mechanistic convergence is notable. This broader pattern is also consistent with skullcap-focused pharmacological studies that characterize its root flavones as pleiotropic regulators of inflammation, oxidative stress, and cancer-relevant signaling networks rather than narrowly acting cytotoxins [190,191].

One of the most translationally relevant GC-specific observations is the reported ability of wogonin to synergize with low-dose oxaliplatin in BGC-823 gastric cancer cells, leading to enhanced apoptosis accompanied by increased JNK phosphorylation at Thr183/Tyr185. This finding is particularly important in the context of combination therapy because oxaliplatin remains a clinically established component of GC treatment, while cumulative neurotoxicity often limits dose intensity. Accordingly, a flavone-assisted strategy that preserves antitumor activity under lower oxaliplatin exposure is mechanistically attractive, but it remains strictly preclinical and model-dependent at present. The same body of work also identifies wogonin, baicalein, acacetin, moslosooflavone, and oroxylin A as candidate GC-relevant components in network pharmacology analyses, with predicted enrichment of PI3K/Akt, p53, and VEGF-related pathways. However, such associations should be interpreted as hypothesis-generating rather than confirmatory, since pathway-level validation in GC-relevant biological systems remains necessary before these predictions can be considered mechanistic evidence [190,191,196].

Gastric precancerous lesion models also indirectly support a chemopreventive rationale. In a network-pharmacology-guided animal study of the multicomponent formulation Huazhuojiedu decoction containing *Radix Scutellariae baicalensis*, treatment improved histopathological features in a rat model of chronic atrophic gastritis and was associated with suppression of increased AKT1 expression, identified as a core hub target in the network analysis. Although this type of study does not allow attribution of activity to *S. baicalensis* alone, it places skullcap root within a GC-relevant preclinical setting involving modulation of proliferation- and apoptosis-related signaling in a recognized precancerous gastric condition [200]. Additional support for this chemopreventive framing comes from extraction and activity studies showing that *Scutellaria* extracts, particularly those derived from *S. baicalensis* roots, exhibit strong antioxidant capacity and anti-inflammatory activity in vitro, as evidenced by FRAP- and DPPH-based radical-scavenging assays and lipoxygenase inhibition. These activities are also method-sensitive, with 70% ethanol under defined microwave-assisted extraction conditions yielding higher phenolic content and stronger in vitro activity in the tested system [201]. In parallel, broader biochemical studies classify *S. baicalensis* among Chinese herbal medicines associated with marked inhibition of lipid peroxidation and eicosanoid production, thereby providing a mechanistic bridge between traditional “heat-clearing/detoxifying” usage and contemporary inflammatory-oxidative models relevant to carcinogenesis risk biology [202].

Additional supportive, though not GC-exclusive, mechanisms further strengthen the biological plausibility of skullcap-derived activity. Studies on traditional Chinese medicine in oncology describe *S. baicalensis* as an herb whose anticancer effects are mediated, at least in part, by inhibiting tumor vascularization [203]. This observation aligns with VEGF-related hypotheses from GC-focused network pharmacology studies, although direct validation in gastric tumor models remains limited [196,203]. Other mechanistic studies indicate that principal skullcap flavones, including baicalin, baicalein, wogonin, and wogonoside, modulate canonical oncogenic and inflammatory pathways such as NF-κB and MAPK, while oroxylin A has been reported to enhance apoptotic sensitivity to fluoropyrimidines in non-gastric tumor systems [192]. Collectively, these findings support a broader interpretation in which skullcap flavones may act as signaling sensitizers and apoptosis-priming agents, a concept relevant to GC chemosensitization [192,196].

Among currently available preclinical studies, extraction parameters and standardization descriptors are better defined than GC-specific dose ranges. Methodological studies clearly show that solvent composition and extraction conditions influence total phenolic yield and antioxidant/anti-inflammatory activity, with root-derived extracts generally outperforming aerial-part extracts under comparable conditions [201]. Likewise, cultivation variables such as light spectrum can alter the baicalein/baicalin and wogonin/wogonoside balance, thereby modifying the chemical exposure delivered by nominally similar skullcap preparations [194]. For purified-compound studies, practical supply constraints may also affect reproducibility, particularly for wogonin, whose low natural abundance has motivated hydrolysis-based preparation from more abundant root-derived precursors [197]. Taken together, these observations indicate that any synthesis of mechanisms relevant to GC should explicitly report plant part, extraction solvent, and parameters, and provide quantitative characterization of the major flavones, because otherwise cross-study comparisons of efficacy, chemosensitization, or apparent synergy are likely to remain substantially confounded [194,199,201].

#### 3.8.3. *Scutellaria baicalensis*: Overall Assessment

Overall, the available preclinical evidence supports *Scutellaria baicalensis* root (Huang-Qin) as a flavone-rich medicinal plant with plausible anticancer and chemopreventive relevance to gastric carcinogenesis. In GC-oriented experimental contexts, reported activities include inhibition of tumor cell proliferation, induction of cell death programs, and enhancement of chemosensitivity, with the most translationally salient finding being wogonin’s ability to sensitize BGC-823 gastric cancer cells to low-dose oxaliplatin via a JNK phosphorylation-associated apoptotic response [196]. Chemopreventive plausibility is further supported by the inclusion of *Radix Scutellariae* in multicomponent interventions that improve histopathology and modulate AKT1 signaling in chronic atrophic gastritis models [200]. In parallel, optimized root extracts display robust antioxidant and anti-inflammatory activity, consistent with broader evidence linking skullcap to suppression of lipid peroxidation and eicosanoid-related inflammatory processes that may be relevant to carcinogenesis risk biology [201,202].

Safety considerations are generally encouraging, but not unequivocal. Clinical-oriented integrative medicine sources describe *S. baicalensis* as having relatively limited known toxicity at therapeutic oral doses of approximately 2–6 g/day, while also documenting rare adverse events, including pneumonitis and hematologic effects in certain administration settings [204]. Likewise, studies focusing on baicalin and wogonin emphasize relatively low adverse-effect profiles in the contexts examined, while simultaneously underscoring that most anticancer claims remain preclinical and therefore require rigorous clinical validation before therapeutic conclusions can be drawn [118,205].

From a translational perspective, three priorities appear particularly important for GC-oriented research. First, standardization remains essential because flavone composition varies across plant parts, cultivation conditions, and extraction methods, with the root consistently the most flavone-rich medicinal input [193,194,201]. Second, pharmacokinetic realism must be addressed because baicalin and related skullcap flavones exhibit formulation-dependent pharmacokinetics and limited bioavailability, which may complicate extrapolation from in vitro potency to in vivo efficacy and justify delivery optimization strategies and more careful evaluation of within-extract pharmacokinetic interactions [118,191,199,206]. Third, mechanistic claims require validation in GC-appropriate systems because pathway predictions derived from network pharmacology do not replace direct experimental confirmation, and because the strongest synergy-relevant observation currently available, namely wogonin plus oxaliplatin in BGC-823 cells, still requires extension to in vivo GC models and clinically plausible exposure conditions before it can be interpreted as more than a promising preclinical hypothesis [196].

Among the plants reviewed here, *Scutellaria baicalensis*, particularly its constituents wogonin and baicalein, currently shows some of the strongest and best-characterized mechanistic preclinical evidence of chemotherapy-modifying activity in gastric cancer.

Table 1 provides a structured overview of preclinical studies investigating selected medicinal plants in gastric cancer, highlighting the experimental models, treatment parameters, and principal antitumor effects, while facilitating comparison of the current evidence base and its translational relevance.

**Figure 1:**
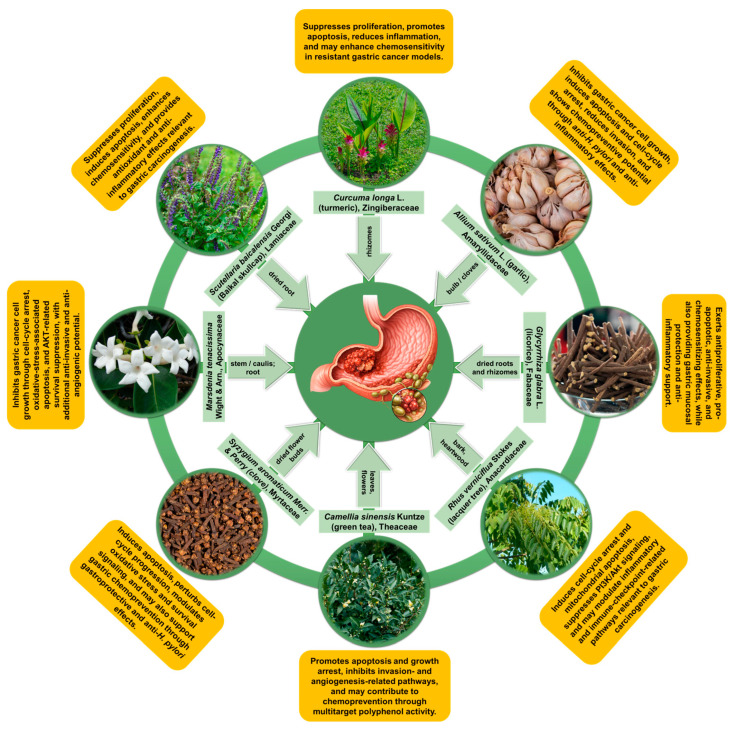
Schematic representation of the eight selected medicinal plants discussed in Section 3 and their main preclinical benefits in gastric cancer. The figure highlights the plant species, the medicinal parts most commonly used, and the principal reported antitumor and chemopreventive effects, including inhibition of proliferation, induction of apoptosis, suppression of invasion- and angiogenesis-related pathways, modulation of inflammation and oxidative stress, and enhancement of chemosensitivity. Sources for the images: *Curcuma longa* (https://www.alamy.com/turmeric-or-curcumin-plant-curcuma-longa-of-the-ginger-family-image395631701.html?imageid=C197D5F2-D7FA-4DD9-A1DD-C1F535928A37&pn=1&searchId=d614d188a0418ddc03b8845eb8e20b9e&searchtype=0, accessed on 30 March 2026), *Allium sativum* (https://www.alamy.com/knoblauch-allium-sativum-markt-markthalle-mercado-dos-lavradores-funchal-insel-madeira-portugal-image560109892.html, accessed on 30 March 2026), *Glycyrrhiza glabra* (https://www.alamy.com/liquorice-roots-close-up-detail-image240196536.html, accessed on 30 March 2026), *Rhus verniciflua* (https://easyscape.com/species/Toxicodendron-vernicifluum%28Chinese-Lacquer-Tree%29, accessed on 30 March 2026), *Camellia sinensis* (https://www.alamy.com/stock-photo-tea-camellia-sinensis-syn-c-thea-fru019419-113499133.html?imageid=C618D021-2B80-4044-A03F-6599A7B20D0C&pn=1&searchId=8196520b7d2af31fe55e77410a4e83b7&searchtype=0, accessed on 30 March 2026), *Syzygium aromaticum* (https://www.alamy.com/cloves-in-a-wooden-bowl-dried-aromatic-flower-buds-of-the-tree-syzygium-aromaticum-used-as-spice-in-cigarettes-and-to-create-a-pomander-image383454853.html, accessed on 30 March 2026), *Marsdenia tenacissima* (https://en.wikipedia.org/wiki/Marsdenia, accessed on 30 March 2026), *Scutellaria baicalensis* (https://www.alamy.com/stock-photo-scutellaria-baicalensis-baikal-skullcap-blue-flower-flowers-flowering-127911855.html?imageid=596C03FE-329F-4D29-9685-4AF651127C37&pn=1&searchId=5fd83f110cd269abe3b4286c39c71ee6&searchtype=0, accessed on 30 March 2026).

Figure 1 schematically illustrates the eight selected medicinal plants discussed in Section 3, together with their main preclinical antitumor and chemopreventive effects in gastric cancer.

## 4. Synergistic Effects Between Natural Compounds and Chemotherapy in Preclinical Gastric Cancer Models

In preclinical oncology, the term “synergy” should be used cautiously. Strictly defined, formal pharmacological synergy requires quantitative evidence that the observed effect of a combination exceeds the effect expected from the individual agents, within an established analytical framework such as the combination index, isobologram, Bliss, Loewe, or related models. By contrast, many preclinical studies report improved chemotherapy response in the presence of a phytocompound without formal combination analysis. Such findings are more appropriately described as chemosensitization rather than as confirmed synergy [118,119,138,218,219].

To improve conceptual clarity, the evidence in this section is categorized into three types: (i) formal synergy demonstrated by quantitative combination analysis; (ii) chemosensitization without formal synergy analysis; and (iii) general anticancer activity without direct evidence of chemotherapy interaction. This distinction is particularly important in gastric cancer, where treatment resistance is multifactorial and apparent enhancement of chemotherapy response may reflect pharmacodynamic sensitization, altered drug metabolism or transport, model-specific vulnerability, or simple additive coactivity rather than true pharmacological synergy [118,119,180,219,220].

In the gastric cancer-focused literature reviewed here, the strongest evidence supports chemosensitization rather than formally demonstrated synergy, as many studies report enhanced apoptosis, reduced viability, or restored drug sensitivity without conducting quantitative combination analyses. Accordingly, throughout this section, the term “synergy” is reserved for studies that explicitly provide formal combination analyses. In contrast, studies lacking such analyses are described as chemosensitization or as combination-associated enhancement of chemotherapy activity.

Consistent with the scope of this review, the discussion below focuses on the eight medicinal plants prioritized in Section 3 and emphasizes both the reported biological interaction and the methodological strength of the supporting evidence.

The most direct preclinical evidence concerns curcumin from *Curcuma longa* and flavones from *Scutellaria baicalensis*, particularly wogonin and baicalein, which have been linked to enhanced chemotherapy response in named GC cell models through mechanisms involving NF-κB suppression, modulation of 5-FU metabolism, and downregulation of multidrug resistance determinants [219,220]. By contrast, for the remaining six plants, the currently available literature more often supports indirect relevance, based either on single-agent anticancer activity in GC, on evidence of chemosensitization in non-gastric models, or pharmacokinetic interaction potential that could be clinically meaningful but has not yet been adequately tested in GC-specific combination systems [118,119,152,158,180,221,222,223,224,225].

Among the plant-derived compounds considered in this review, curcumin provides one of the clearest GC-specific examples of combination activity with conventional chemotherapy. In SGC-7901 gastric cancer cells, curcumin co-administered with the topoisomerase inhibitors etoposide and doxorubicin was reported to suppress NF-κB activation further and downregulate downstream anti-apoptotic mediators, including Bcl-2 and Bcl-xL, thereby promoting apoptosis and reversing chemoresistance [219]. This interpretation is consistent with broader evidence indicating that phytochemicals can function as multitarget chemosensitizers by shifting the balance from pro-survival signaling toward apoptotic cell death [139,218].

Curcumin is also widely discussed in the interaction literature as a potential pharmacokinetic bioenhancer that can modulate drug-metabolizing enzymes and efflux transporters, including P-glycoprotein [180]. Studies focused on the clinical relevance of natural product–anticancer treatment interactions similarly identify *Curcuma longa* as a botanical with documented potential for interactions when administered concomitantly with cancer therapy [118]. Such effects may theoretically be beneficial if they increase intratumoral chemotherapy exposure, but may also be detrimental if they increase systemic toxicity or unpredictably alter clearance [118,119,180].

For this reason, GC combination studies attributing benefit primarily to NF-κB suppression should ideally also quantify intracellular chemotherapy accumulation and assess the expression or activity of relevant transporters and metabolic enzymes. Without such analyses, it remains difficult to distinguish true pharmacodynamic chemosensitization from pharmacokinetically mediated changes in drug exposure [118,119,180]. This issue represents a recurrent translational pitfall in interpreting curcumin-based combination strategies.

Within the eight plants prioritized in this review, *Scutellaria baicalensis* provides the most explicit GC-model evidence for chemosensitization. Wogonin has been reported to increase the susceptibility of MGC803 gastric cancer cells to 5-FU-induced apoptosis. Mechanistically, this effect is particularly noteworthy because it appears to involve both suppression of a canonical pro-survival pathway and modulation of a chemotherapy-specific metabolic determinant. More specifically, wogonin was reported to inhibit NF-κB nuclear translocation and IκB phosphorylation, and to downregulate dihydropyrimidine dehydrogenase (DPD), thereby slowing 5-FU metabolism and enhancing the efficacy of low-dose 5-FU [219].

This dual mechanism is translationally attractive because it targets two major classes of resistance-related processes in GC: activation of survival signaling pathways and reduced drug effectiveness during treatment exposure [180,219].

Hypoxia is a major determinant of therapeutic response in GC and may substantially alter the activity of both cytotoxic drugs and adjunctive phytocompounds. In this context, baicalein has been reported to increase the sensitivity of gastric cancer cells to 5-FU under hypoxic conditions [219].

From a translational standpoint, this evidence highlights the need for future synergy studies to incorporate hypoxia-aware experimental design, including oxygen control, assessment of hypoxia-associated markers, and matched normoxic comparators. Without these design features, the relevance of apparently positive combination effects may be difficult to interpret in light of the biological reality of gastric tumors, which commonly contain heterogeneous hypoxic regions [219].

A GC-focused analysis of Nrf2 biology further indicates that baicalein downregulated MDR1 expression in both SGC-7901 and cisplatin-resistant SGC-7901/DDP cells in a dose- and time-dependent manner, and this finding was discussed in relation to the restoration of cisplatin responsiveness. Importantly, the same synthesis noted that baicalein also increased Nrf2 expression and decreased Keap1 levels, indicating antioxidant activity, but concluded that these redox-related changes did not explain the observed enhancement of cisplatin sensitivity in the resistant model [220].

This mechanistic nuance is particularly relevant because Nrf2 has context-dependent functions in GC and is frequently associated with increased antioxidant capacity, chemoresistance, and poor prognosis when persistently activated [220]. The baicalein data therefore suggest that redox pathway modulation may accompany, but does not necessarily mediate, chemosensitization. Instead, transporter-related mechanisms, such as MDR1 downregulation, may be more proximate drivers of restored drug sensitivity in certain resistant GC settings [152,220]. More broadly, studies on plant-derived resistance modulators support the plausibility of such transporter-centered mechanisms, particularly when combined with induction of cell death programs and attenuation of drug resistance phenotypes [152].

*Glycyrrhiza glabra* is repeatedly described as a bioactive-rich medicinal plant with properties relevant to oncology, including immunomodulatory and signaling-related effects associated with glycyrrhizin and related constituents [221]. Studies report that glycyrrhizin can enhance the effects of chemotherapeutic drugs, such as paclitaxel, in cancer cells [180].

*G. glabra* contains constituents reported to modulate CYP3A4 activity, thereby raising the possibility of pharmacokinetic interactions with anticancer drugs metabolized through CYP-dependent pathways [180]. The broader herb–drug interaction literature emphasizes that concomitant use of natural products can lead to clinically relevant outcomes, whether beneficial or adverse, depending on whether exposure to the anticancer drug is increased, decreased, or unchanged [118,119,180,221].

Because taxanes remain part of the broader chemotherapeutic landscape discussed in GC resistance-related literature, the principal implication of the garlic evidence in the present context is cautionary rather than synergistic [219]. Garlic-derived products may alter chemotherapy metabolism and systemic exposure, potentially confounding apparent pharmacodynamic synergy and increasing toxicity risk if not carefully controlled [118,119,180]. This interpretation is fully consistent with herb–drug interaction frameworks that emphasize the anticipation and monitoring of botanical co-medication during chemotherapy, since both harmful and beneficial outcomes are possible depending on the magnitude and direction of changes in exposure [118,119].

Green tea polyphenols, especially epigallocatechin-3-gallate (EGCG), are widely discussed as chemosensitizers that act synergistically with multiple anticancer agents across experimental systems, including platinum-based drugs [152]. Studies have linked EGCG-mediated enhancement of cisplatin sensitivity to increased intracellular platinum accumulation and greater cisplatin–DNA adduct formation. These effects are associated with upregulation of the copper transporter CTR1 and are supported by xenograft evidence in cancer [152].

Clove-derived eugenol is described in the literature as an anticancer and chemopreventive phytocompound with particular relevance to gastric biology. Notably, eugenol has been reported to reduce tumor incidence and burden in an N-methyl-N′-nitro-N-nitrosoguanidine-induced rat model of gastric carcinogenesis and to suppress invasion- and angiogenesis-related processes in that setting [222]. Additional studies on spices and spice-derived phytochemicals similarly support the anticancer relevance of clove and eugenol within broader chemopreventive and therapeutic frameworks [223,224].

Mechanistic discussions place *M. tenacissima* within broader herb–drug interaction frameworks that involve CYP3A4 inhibition and transporter-mediated resistance modulation, including effects on MRP2 [119,225].

In SGC-7901 cells, curcumin enhanced the effects of etoposide and doxorubicin in association with reduced NF-κB activation and downregulation of anti-apoptotic targets such as Bcl-2 and Bcl-xL [219]. In MGC803 cells, wogonin likewise increased susceptibility to 5-FU-induced apoptosis while suppressing NF-κB nuclear translocation and IκB phosphorylation [219]. This convergence is consistent with broader evidence indicating that natural compounds frequently enhance chemotherapy responses through multitarget pro-apoptotic reprogramming rather than through single-pathway blockade alone [139,218].

A major translational caveat, however, is that NF-κB-centered interpretations are often based on pathway activity readouts without parallel assessment of intracellular chemotherapy exposure. As emphasized in herb–drug interaction analyses, the observed interaction phenotype may reflect not only pharmacodynamic sensitization but also pharmacokinetic changes that alter tumor-cell drug accumulation [118,119,180].

Wogonin enhanced 5-FU activity in MGC803 cells by downregulating DPD, suggesting that impaired 5-FU catabolism may contribute to improved efficacy at low doses [219]. Second, baicalein was linked to dose- and time-dependent downregulation of MDR1 in both parental and cisplatin-resistant GC cells, supporting the interpretation that transporter modulation may help restore cisplatin responsiveness in resistant settings [220]. These mechanisms are particularly important because they sit at the interface of pharmacodynamics and pharmacokinetics. While reduced DPD or MDR1 activity may enhance tumor sensitivity, the broader literature on interactions makes clear that modulation of enzymes and transporters can also alter systemic exposure and toxicity [118,119,180]. For this reason, future GC combination studies investigating DPD- or MDR1-related mechanisms should combine pathway-level biomarkers with direct exposure readouts, including intracellular drug quantification whenever feasible [118,119,180].

Future studies should prioritize resistant and context-stressed GC models, including drug-resistant derivatives and hypoxia-adapted systems, because these settings more closely mirror the biological conditions under which adjuvant chemosensitization would be clinically meaningful [219,220]. Additionally, explicit dose–response matrices and clearly reported concentrations are essential, as the lack of quantitative detail remains a pervasive limitation in secondary summaries and substantially undermines reproducibility and interpretability [118,119]. Pharmacodynamic endpoints should be evaluated alongside pharmacokinetic readouts, including intracellular drug accumulation and enzyme/transporter profiling, to avoid misclassifying exposure-driven effects as pathway-driven synergy [118,119,180]. Translational success depends as much on experimental rigor and mechanistic resolution as on the initial observation of a favorable combination effect [118,119,180,219,220].

To provide a structured synthesis of the evidence and avoid overinterpretation, Table 2 summarizes the relevant preclinical literature into three evidence categories: formal synergy demonstrated by quantitative combination analysis, chemosensitization without formal synergy analysis, and general anticancer activity without direct evidence of a chemotherapy interaction.

Overall, the currently available gastric cancer-focused literature more often supports chemosensitization than rigorously demonstrated formal synergy, and this distinction should be kept in mind when interpreting apparently favorable combination effects. These considerations also support the need for a more operational translational framework to guide future preclinical prioritization in this field.

### A Proposed Translational Evidence Ladder for Future Preclinical Studies

To improve translational rigor in this field, future studies evaluating medicinal plant bioactives in combination with standard chemotherapy for gastric cancer should ideally follow a stepwise evidence ladder rather than relying solely on isolated positive findings from simplified models. At a minimum, the first level should include two-dimensional mechanistic screening in well-characterized gastric cancer cell lines, using clearly defined phytochemical preparations, dose–response matrices, and formal evaluation of whether the observed interaction reflects true pharmacological synergy, chemosensitization, or only additive coactivity. At this stage, mechanistic endpoints such as apoptosis, cell-cycle effects, oxidative stress, transporter regulation, EMT markers, and survival signaling may be used to identify biologically plausible interaction patterns. Still, such findings should be interpreted as preliminary.

The second level should include validation in more demanding in vitro systems that better reflect treatment resistance and biological context. These may include drug-resistant derivatives, hypoxia-adapted models, stemness-enriched systems, or coculture settings that incorporate stromal influences. This step is important because an interaction that appears favorable in treatment-naïve monolayer cultures may weaken, disappear, or even become antagonistic in resistant or microenvironmentally protected settings. Accordingly, combinations intended for translational prioritization should demonstrate activity not only in reductionist screening models but also in biologically more challenging systems relevant to gastric cancer chemoresistance.

The third level should involve advanced, patient-relevant, three-dimensional platforms, including spheroids, organoids, patient-derived organoids, assembloids, or other biomimetic coculture systems. These models provide a more realistic representation of tumor architecture, intratumoral heterogeneity, and stromal protection. They are therefore better suited to testing whether phytochemical-mediated enhancement of chemotherapy persists under conditions that more closely resemble human disease. Where feasible, biomarker-oriented analyses should also be incorporated at this stage to identify the molecular or phenotypic contexts in which a given combination is most likely to remain effective.

The fourth level should consist of in vivo validation in models that are informative for both efficacy and exposure, ideally including xenografts, patient-derived xenografts, or other clinically relevant preclinical systems. At this stage, efficacy assessment should be paired with pharmacokinetic-informed evaluation, including dose justification, formulation considerations, systemic and, where feasible, intratumoral exposure, and potential herb–drug interactions affecting chemotherapy disposition or toxicity. Without this layer of PK-informed validation, apparent in vitro promise cannot be considered sufficiently mature for meaningful translational prioritization.

Overall, strong translational claims should be reserved for combinations that coherently progress across this ladder. Positive results observed only in standard two-dimensional systems may still be useful for generating mechanistic hypotheses. Still, they should not be interpreted as equivalent to evidence supported by resistant-cell validation, advanced three-dimensional or patient-derived models, and in vivo pharmacology-aware confirmation.

As a practical minimum, future studies should not claim strong translational relevance unless a candidate combination has been evaluated in at least one resistant/context-relevant in vitro model, one advanced three-dimensional or patient-derived platform, and one in vivo model with PK-aware exposure assessment. Consistent reporting of concentration ranges, exposure duration, comparator groups, mechanistic endpoints, and the analytical basis for assigning synergy should be considered a minimum reporting expectation in future gastric-cancer combination studies.

## 5. Conclusions, Limitations, and Future Perspectives

The evidence synthesized in this review supports medicinal plant bioactives as biologically plausible adjuncts to gastric cancer therapy, particularly because they can simultaneously target multiple resistance-associated processes rather than acting through a single cytotoxic mechanism. Across the eight selected plants, the most consistently reported GC-relevant effects included inhibition of proliferation, induction of apoptosis, suppression of invasion- and angiogenesis-related pathways, modulation of oxidative stress and inflammatory signaling, and attenuation of multidrug-resistance-associated phenotypes. This multitarget pharmacology is especially relevant in gastric cancer, where treatment failure is rarely driven by a single molecular abnormality and more commonly reflects the interaction of tumor heterogeneity, microenvironmental influences, adaptive stress responses, and treatment-induced selection pressure.

At the same time, the reviewed literature does not support treating all medicinal plants or all phytochemical–chemotherapy combinations as equally substantiated. The strongest and most mechanistically well-developed preclinical evidence currently concerns curcumin from *Curcuma longa* and flavones from *Scutellaria baicalensis*, especially wogonin and baicalein, which have been linked to enhanced chemotherapy response through mechanisms including NF-κB suppression, modulation of 5-fluorouracil metabolism, and downregulation of multidrug resistance determinants. Additional GC-model combination studies further support the chemosensitizing potential of garlic extracts with doxorubicin; EGCG with 5-fluorouracil in resistant models; eugenol-based systems with cisplatin; and Marsdenia tenacissima-derived preparations with S-1. Liquiritin also showed compelling resistance-reversal activity in TRAIL-based models, although this setting lies somewhat outside the strict framework of standard cytotoxic chemotherapy. By contrast, for other plants, especially Rhus verniciflua, the current evidence remains more supportive of general anticancer activity than of directly demonstrated GC-specific chemotherapy synergy.

Collectively, these findings suggest that medicinal plant bioactives should not be viewed merely as empirical “natural add-ons” to chemotherapy, but rather as candidate modulators of drug response, with value that depends on chemical definition, mechanistic specificity, and experimental context. In their most promising form, such compounds may help reduce the effective dose of conventional chemotherapy, restore sensitivity in resistant disease, or widen the therapeutic window of established regimens. However, these possibilities remain preclinical and should be viewed as translational opportunities rather than clinically validated strategies.

Despite the growing number of preclinical studies, several limitations substantially weaken the translational weight of the current literature. First, the chemical definition of the tested interventions is often inadequate. Many studies use broad terms such as “extract,” “fraction,” “tea,” “garlic extract,” or “skullcap flavones” without sufficiently detailed reporting of plant source, medicinal part, extraction conditions, constituent quantification, batch variability, or marker compounds. This creates a major reproducibility problem, because apparently similar botanical interventions may in fact represent very different chemical exposures.

Second, the interpretation of “synergy” is frequently methodologically weak. In many reports, synergy is inferred from greater activity of the combination than of either single agent alone, but without formal pharmacological analyses capable of distinguishing true synergy from additivity, co-activity, or exposure-driven sensitization. This issue is compounded by incomplete concentration reporting, inconsistent comparator design, and the common absence of dose–response matrices. As a result, some published combination effects remain difficult to benchmark across studies or to interpret in relation to clinically achievable exposure.

Third, the dominant preclinical models remain limited in translational realism. A large proportion of the literature relies on conventional two-dimensional cell culture systems, which do not adequately reproduce the architecture, stromal context, immune interactions, or metabolic gradients of gastric tumors. Even when in vivo xenograft studies are available, they often remain reductionist and may not capture the biological heterogeneity that defines treatment response in human gastric cancer. This limitation is particularly important for phytochemical–chemotherapy combinations, because context-dependent effects may differ substantially between simplified systems and more physiologically relevant models.

Fourth, pharmacokinetic and safety considerations remain insufficiently integrated into efficacy claims. Many phytocompounds discussed in this review, including curcumin, EGCG, and skullcap flavones, are affected by poor bioavailability, formulation dependence, or uncertain tissue exposure. In parallel, several medicinal plants may cause herb–drug interactions by modulating transporters, metabolic enzymes, or redox pathways. Consequently, an apparently favorable in vitro interaction may reflect altered drug exposure, altered toxicity risk, or both, rather than purely pharmacodynamic synergy. This risk is especially important in gastric cancer, where standard chemotherapies already have narrow therapeutic windows and dose-limiting toxicities.

Finally, the evidence base remains highly uneven across plants and compounds. Multiple mechanistic and combination studies support some interventions, whereas others are represented only by single reports, indirect rationale, or non-gastric analogies. This asymmetry means the field should not be presented as uniformly mature. Instead, it is more accurate to conclude that gastric cancer currently offers a small number of promising plant–drug combinations, embedded within a much broader yet still methodologically fragmented exploratory literature.

Future progress in this field will depend first on stricter chemical and pharmacological standardization. Studies should clearly define botanical identity, plant part, extraction method, formulation, marker compounds, and quantitative phytochemical composition before attempting mechanistic or synergistic interpretation. For purified compounds, purity, stability, and batch consistency should be explicitly reported. For multicomponent preparations, the major constituents most likely to drive biological activity should be quantified and linked to experimental outcomes whenever possible. Without this level of chemical precision, the translational development of plant-derived adjuncts for gastric cancer will remain fundamentally unstable.

A second priority is adopting a more rigorous combination-study design. Future work should move beyond simple “combination versus single-agent” comparisons and incorporate full dose–response matrices, formal synergy analyses, and orthogonal validation strategies. In addition, pharmacodynamic endpoints should be paired with pharmacokinetic readouts, including intracellular drug accumulation, transporter expression, enzyme activity, and, where feasible, tumor-level exposure measurements. This is particularly important for compounds that may act as bioenhancers or transporter modulators, as their apparent benefit may otherwise be misclassified.

Third, the next generation of studies should make broader use of clinically relevant GC models. Drug-resistant derivatives, hypoxia-adapted systems, patient-derived organoids, patient-derived xenografts, assembloids, and more advanced three-dimensional co-culture platforms are likely to be far more informative than standard monolayer cell lines alone. Such models would allow more realistic testing of whether phytochemical-mediated chemosensitization persists in the presence of stromal protection, microenvironmental stress, or intratumoral heterogeneity. They would also support biomarker-oriented research to identify which tumors are most likely to benefit from specific plant-derived adjuncts.

A fourth major direction concerns the optimization of formulation and delivery. Because poor oral bioavailability and unstable systemic exposure remain recurring barriers for several high-interest phytocompounds, formulation science should be integrated early rather than treated as a late-stage technical correction. Nanoformulations, carrier-based delivery systems, chemically optimized derivatives, and rational route selection may all help bridge the gap between in vitro potency and in vivo feasibility. However, such approaches should not merely increase exposure; they should also be evaluated for altered toxicity, tissue distribution, and drug–drug interaction potential.

Finally, clinical translation will require a more disciplined approach to prioritization. Rather than dispersing effort across many poorly characterized botanicals, the field would benefit from first focusing on combinations with the strongest mechanistic and preclinical support for GC. At present, these include curcumin-based combinations, skullcap-derived flavones such as wogonin and baicalein, EGCG in resistant fluoropyrimidine settings, eugenol-based strategies to sensitize cisplatin, and selected preparations from *Marsdenia tenacissima*. Early-phase translational studies should be designed not only to test efficacy signals but also to establish pharmacokinetic plausibility, safety boundaries, herb–drug interaction profiles, and biomarker-informed patient selection. Only through this stepwise, pharmacology-aware approach can medicinal plant bioactives move from promising preclinical observations toward credible adjunctive strategies in gastric cancer management.

Future progress in this area will depend not only on identifying favorable plant–drug combinations but also on testing them along a stepwise translational evidence ladder that spans mechanistic 2D screening, resistant and 3D systems, and, finally, in vivo PK-informed validation.

## Figures and Tables

**Table 1:** Overview of relevant preclinical studies evidencing the main outcomes of selected medicinal plants in gastric cancer treatment.

Plant, Used Parts, Family	Extract Type	Phytocompounds	Gastric Cancer Cell Line	Dosage	Outcomes	Ref.
*Curcuma longa* L. Rhizomes Zingiberaceae	—	Curcumin	BGC-823; SGC-7901; MKN-28	Curcumin 0–200 µM for 24, 48, and 72 h in viability assays; mechanistic assays were mainly conducted after 48 h using 5–15 µM in BGC-823 and 5–20 µM in SGC-7901/MKN-28 cells; in co-treatment experiments, 3-methyladenine (3-MA) was used at 10 mM.	Curcumin inhibited cell viability in a time- and dose-dependent manner (24 h IC_50_: 37.58 µM in BGC-823, 50.45 µM in SGC-7901, and 16.17 µM in MKN-28; 48 h IC_50_: 15.18, 18.53, and 15.84 µM, respectively). It induced apoptosis, evidenced by increased TUNEL positivity and Annexin V-positive cells, downregulation of Bcl-2, upregulation of Bax, and activation of caspase-3/-9. Curcumin also triggered autophagy, as shown by AVO formation, LC3-I to LC3-II conversion, and increased Beclin1, Atg7, and Atg5-Atg12 levels, while suppressing the PI3K/Akt/mTOR/p70S6K pathway. Co-treatment with 3-MA enhanced curcumin-induced apoptosis and further reduced viability, indicating that curcumin-induced autophagy was protective.	[207]
*Allium angulosum* L. Bulb/cloves Amaryllidaceae	65% ethanol extract (24 h maceration)	—	MKN28, MKN74	0.3 μg/mL for 24 and 48 h	Showed cytotoxicity in both cell lines, more markedly in MKN74. In MKN28, viability decreased to 75.2% at 24 h and 48.6% at 48 h; in MKN74, to 40.1% at 24 h and 34.9% at 48 h. No clear CDH1/COX2-modulating effect was reported.	[208]
*Allium sativum* L. (Poland) Bulb/cloves Amaryllidaceae	65% ethanol extract (24 h maceration)	—	MKN28, MKN74; DOX co-treatment evaluated in MKN74	0.3 μg/mL for 24 and 48 h; in combination experiments, extract + doxorubicin 1 µM for 24 h	In MKN74, increased CDH1 and decreased COX2 expression, especially after 48 h; COX-2 mRNA remained below control at 48 h. Exhibited cytotoxicity in MKN74 (59.9% viability at 24 h; 65.0% at 48 h), whereas the effect in MKN28 was limited. Showed a synergistic effect with doxorubicin in MKN74, reducing viability to 52.1% in the combination setting.	
*Allium sativum* L. (Malaysia) Bulb/cloves Amaryllidaceae	65% ethanol extract (24 h maceration)	—	MKN28, MKN74; DOX co-treatment evaluated in MKN74	0.3 μg/mL for 24 and 48 h; in combination experiments, extract + doxorubicin 1 µM for 24 h	In MKN74, upregulated CDH1 and downregulated COX-2, with the strongest effect evident after 48 h. Reduced MKN74 viability to 74.4% at 24 h and 71.1% at 48 h, with minimal toxicity in MKN28—also enhanced doxorubicin cytotoxicity in MKN74, with 62.6% viability under co-treatment conditions.	
*Allium ursinum* L. Bulb/cloves Amaryllidaceae	65% ethanol extract (24 h maceration)	—	MKN28, MKN74; DOX co-treatment evaluated in MKN74	0.3 μg/mL for 24 and 48 h; in combination experiments, extract + doxorubicin 1 µM for 24 h	Displayed marked cytotoxicity in both cell lines, particularly in MKN74 (35.3% viability at 24 h; 31.5% at 48 h). In MKN74, it also increased CDH1 and reduced COX-2 expression. In combination with doxorubicin, no loss of cytotoxic efficacy was observed, but a clear synergistic interaction was not demonstrated.	
*Allium tibeticum* Rendle Bulb/cloves Amaryllidaceae	65% ethanol extract (24 h maceration)	—	MKN28, MKN74; DOX co-treatment evaluated in MKN74	0.3 μg/mL for 24 and 48 h; in combination experiments, extract + doxorubicin 1 µM for 24 h	In MKN74, increased CDH1 and decreased COX2 at both mRNA/protein levels, with more evident protein changes after 48 h. Cytotoxicity was moderate in MKN74 (74.3% viability at 24 h; 84.6% at 48 h) and limited in MKN28. In co-treatment experiments, it did not reduce doxorubicin activity.	
*Allium lusitanicum* Lam. Bulb/cloves Amaryllidaceae	65% ethanol extract (24 h maceration)	—	MKN28, MKN74	0.3 μg/mL for 24 and 48 h	Produced moderate cytotoxicity, particularly in MKN74 (86.9% viability at 24 h; 67.3% at 48 h) and milder effects in MKN28. No consistent modulation of CDH1/COX2 expression was reported.	
*Glycyrrhiza glabra* L. Dried roots and rhizomes Fabaceae	Isolated compound from the hydrophobic fraction of licorice root	Glabridin (isoflavane/isoflavonoid)	MKN-45	Glabridin 6–40 µM for 48 h in proliferation assays; 25 µM glabridin was mainly used in mechanistic experiments, alone or combined with 5-FU (0.1 mM), for 3 days in apoptosis/caspase assays, 48–72 h in invasion assays, and 10 days in colony formation assays.	Glabridin exerted dose- and time-dependent antiproliferative effects, reduced colony formation, induced apoptosis (~55.4%), activated caspase-3, -8, and -9, and inhibited invasion (~40.96% invading cells vs. 69.67% in controls). It also modulated apoptosis- and invasion-related markers, including decreased Bcl-2, EGFR, MMP-2, and p16, with increased Bax and altered cadherin signaling. In combination with 5-FU, glabridin further enhanced the antitumor effect, markedly increasing apoptosis (~93.9%) and reducing invasion (~15.23% of invading cells), while also affecting the expression of cyclin D1, Ki-67, MMP-9, and VEGF.	[209]
*Rhus verniciflua* Stokes Bark; heartwood/lignum Anacardiaceae	Allergen-free refined extract (RVSE); hot-water extract followed by 95% ethanol refining	Fustin and fisetin (major quantified flavonoids)	AGS	Cell viability: 0–150 μg/mL for 24 h; migration/invasion and mechanistic assays: 0–100 μg/mL for 24 h	RVSE showed no significant cytotoxicity at 25–100 μg/mL, but dose-dependently suppressed AGS cell migration and invasion. At 100 μg/mL, invasion was reduced by 52.7 ± 3.2%. Mechanistically, RVSE downregulated MMP-9 and uPA, markedly upregulated PAI-1 (443 ± 60% at 100 μg/mL), and inhibited STAT3 phosphorylation, supporting an anti-metastatic effect in gastric cancer cells.	[210]
*Camellia sinensis* (L.) Kuntze Leaves, flower buds, Theaceae	Methanol extract from the dried flower buds	Chakasaponin I	MKN-45	IC_50_ = 16.8 µM; in mechanistic assays performed in HSC-2 cells, 5–10 µM for 24–48 h were used.	Showed clear antiproliferative activity against MKN-45 cells. In additional non-gastric HSC-2 assays, it induced G2/M arrest and apoptosis, as evidenced by DNA fragmentation and caspase-3/7 activation, suggesting a pro-apoptotic mechanism.	[211]
		Chakasaponin II		IC_50_ = 17.3 µM; in mechanistic assays performed in HSC-2 cells, 10–15 µM for 24–48 h were used.	Exhibited antiproliferative activity in MKN-45 cells. In HSC-2 cells, it promoted S/G2-M phase accumulation and apoptosis, as evidenced by DNA fragmentation and caspase-3/7 activation.	
		Floratheasaponin A		IC_50_ = 4.5 µM; in mechanistic assays performed in HSC-2 cells, 2.5–7.5 µM for 24–48 h were used.	This was the most potent tested tea-flower constituent against MKN-45 cells, showing marked antiproliferative activity. In HSC-2 cells, it also induced cell-cycle arrest and apoptosis, as evidenced by DNA fragmentation and caspase-3/7 activation.	
		(–)-Epigallocatechin-3-O-gallate (EGCG)		IC50 > 100 µM (70.3% cell viability at 100 µM); in HSC-2 mechanistic assays, 25–75 µM for 24–48 h were used.	Showed weak/limited antiproliferative activity in MKN-45 cells in this study. Unlike the saponins, EGCG did not significantly affect cell-cycle distribution or induce apoptosis in HSC-2 cells at the tested concentrations.	
	Extract from the dried leaves	(−)-Epigallocatechin-3-gallate (EGCG)	SGC-7901	In vitro: 5–80 µM for 24 and 48 h in MTT assays; 15, 30, and 45 µM were used in apoptosis/cell-cycle, luciferase, and signaling assays; colony formation was assessed after 72 h. In vivo: 0.5, 1, or 2 mg/kg, intravenous (caudal vein), once every 2 days for 21 days in nude mice bearing SGC-7901 xenografts.	EGCG inhibited SGC-7901 proliferation with IC_50_ values of 28.87 ± 1.46 µM (24 h) and 17.35 ± 1.24 µM (48 h), reduced colony formation, induced apoptosis, and caused G0/G1 cell-cycle arrest. Mechanistically, it suppressed canonical Wnt/β-catenin signaling, as evidenced by reduced levels of p-β-catenin (Ser552), p-GSK3β (Ser9), nuclear β-catenin, and downstream targets (ccnd1, c-myc, c-jun), along with decreased PCNA, cyclin D1, and Bcl-2 expression. In vivo, EGCG significantly inhibited xenograft growth and reduced tumor weight, β-catenin, and PCNA levels, with no apparent toxicity, as evidenced by unchanged body weight.	[212]
*Syzygium aromaticum* (L.) Merr. & L.M.Perry Dried unopened flower buds, Myrtaceae	Crude 80% ethanolic extract from the flower buds	Phenolic compounds	AGS	Various concentrations for 24, 48, and 72 h in MTT assays; reported IC_50_ values were 381.1 μg/mL (24 h), 118.7 μg/mL (48 h), and 116.2 μg/mL (72 h). Apoptosis was assessed after 48 h using 1× IC_50_.	The extract showed time- and dose-dependent antiproliferative activity against AGS cells and was less toxic to normal HDF cells (IC_50_ 649 μg/mL at 48 h). Flow cytometry indicated induction of apoptosis, with a total apoptotic fraction of 21.61%, predominantly early apoptosis. Overall, the study supports an antiproliferative and pro-apoptotic effect of crude clove extract in gastric carcinoma cells.	[213]
*Marsdenia tenacissima* (Roxb.) Wight & Arn. Stem/caulis; occasionally root Apocynaceae	C21 steroid-enriched fraction refined from *M. tenacissima* ethyl acetate extract	C21 steroidal glycosides	BGC-823, SGC-7901, AGS (mechanistic assays were mainly performed in BGC-823 and AGS)	Proliferation assays: 0–40–80–160 μg/mL (RTCA) and 0–20–40–80–160–320 μg/mL for 24 h (MTS). Cell-cycle/apoptosis assays: 80 and 160 μg/mL for 24 h. Mechanistic assays: 120 μg/mL for 24 h, alone or combined with chloroquine (CQ, 20 µM).	The C21 fraction inhibited gastric cancer cell proliferation in a dose-dependent manner, with IC_50_ values of 80.57 μg/mL (BGC-823), 146.3 μg/mL (SGC-7901), and 102.8 μg/mL (AGS). It induced G2/M cell-cycle arrest and apoptosis, increased ROS, SOD, and H_2_O_2_ levels, upregulated cleaved PARP and BAX, and downregulated Bcl-2 and p-AKT. The fraction also promoted autophagy-related changes, including LC3-II accumulation. It increased Beclin-1 and ATG-5, and co-treatment with CQ further enhanced apoptosis, indicating that C21-induced autophagy was protective and that inhibition of autophagy potentiated the pro-apoptotic effect.	[214]
*Scutellaria baicalensis* Georgi Dried root, Lamiaceae	Purified flavonoid compound	Baicalein	SGC-7901, MKN45 (mechanistic and xenograft experiments were mainly performed in SGC-7901)	In vitro: 0–120 µM for 24, 48, and 72 h in MTT assays; 15–60 µM for 48 h in colony formation, apoptosis, and mitochondrial membrane potential assays; 30–120 µM for 48h in cell-cycle assays. In vivo: 15 or 50 mg/kg/day, intragastrically, for 7 days in nude mice bearing SGC-7901 xenografts.	Baicalein inhibited gastric cancer cell growth and colony formation in a dose- and time-dependent manner, induced marked S-phase arrest in SGC-7901 cells, and promoted apoptosis through the mitochondrial pathway, as shown by mitochondrial membrane depolarization, Bcl-2 downregulation, Bax upregulation, reduced pro-caspase-3, and increased cleaved PARP. In vivo, baicalein significantly suppressed SGC-7901 xenograft growth and reduced tumor weight, supporting its antitumor activity in gastric cancer models.	[215]
	Flavonoid extract from Korean *S. baicalensis* Georgi (FSB)	Flavonoid-rich extract containing 16 identified constituents, including pentahydroxyflavanone derivative, pentahydroxyflavanone, viscidulin I-O-diglucoside, pentahydroxyflavone, viscidulin III-O-glucoside, tetrahydroxyflavone, iridin, eriodictyol, puerarin, viscidulin III, baicalin, scutellarein, and isoscutellarein	AGS	0–400 μg/mL for 24 h in viability assays; 50 and 100 μg/mL for 24 h in cell-cycle, apoptosis, mitochondrial membrane potential, caspase-8, and Western blot assays	FSB inhibited AGS cell viability in a concentration-dependent manner, with an IC_50_ of ~100 μg/mL at 24 h. It increased the sub-G1 population (from 7% in controls to 21% and 27% at 50 and 100 μg/mL, respectively), enhanced Annexin V-positive apoptosis, and induced nuclear fragmentation. Mechanistically, FSB triggered mitochondria-mediated apoptosis, as shown by decreased mitochondrial membrane potential, increased Bax/Bcl-xL ratio, reduced procaspase-3 and procaspase-9, and increased cleaved caspase-3 and cleaved PARP; no activation of the extrinsic pathway was observed, as Fas/FasL expression decreased and caspase-8 was not activated.	[216]
	Purified acidic polysaccharide fraction from aqueous extract, followed by ethanol precipitation, DEAE-cellulose ion-exchange chromatography, and gel chromatography; the main active fraction was SBP-A-3-2	Acidic polysaccharides, especially SBP-A-3-2; major monosaccharides: GalA, Rha, Gal, and Ara. SBP-A-3-2 was characterized as an ~8.2 kDa pectic polysaccharide rich in homogalacturonan (HG) with a minor RG-I domain and trace t-β-Gal and →3,5)-α-Ara side chains	AGS, HGC-27 (AGS was the main Gal-8-high responsive model; HGC-27 was used as a lower-Gal-8 comparison line)	For SBP-A subfractions: 0, 1, 2, and 4 mg/mL. Proliferation (MTT): 24, 48, and 72 h; wound-healing: 24 h; Transwell migration: 48 h. The same experimental workflow was then applied to the purified SBP-A-3 subfractions (SBP-A-3-1 and SBP-A-3-2)	The acidic polysaccharide fractions showed anti-gastric adenocarcinoma activity in vitro. In AGS cells, SBP-A-3 was the most active among the initial acidic fractions, inhibiting proliferation by 18%, 49%, and 75% at 24, 48, and 72 h, respectively; it also produced the strongest migration inhibition in wound-healing assays (wound-healing rate 24.42%). After further purification, SBP-A-3-2 was identified as the principal Gal-8-binding fraction and was reported to exert the most pronounced inhibitory effect on AGS-cell proliferation and migration, supporting a Gal-8-targeted anti-gastric adenocarcinoma effect. In HGC-27 cells, antiproliferative/migratory effects were also observed, but the activity pattern differed, with SBP-A-1 being more active than SBP-A-3, suggesting that the HGC-27 response may be less dependent on Gal-8. The authors proposed that SBP-A-3-2 may interfere with Gal-8-related Ras signaling, although this mechanism was inferred rather than directly demonstrated.	[217]

**Table 2:** Structured overview of preclinical evidence on medicinal plant bioactives and chemotherapy interactions in gastric cancer models, classified as formal synergy, chemosensitization without formal synergy analysis, or general anticancer activity without direct evidence of chemotherapy interaction.

Plant, Family	Phytocompound(s)/Type of Extract	Chemotherapy Partner	Gastric Cancer Model	Dosage/Concentration and Exposure Conditions	Main Mechanistic Endpoint(s) + Key Interaction Results	Outcomes	Evidence Classification	Ref.
*Curcuma longa* L. Zingiberaceae	Curcumin	5-Fluorouracil + cisplatin (FP regimen; 5-FU + DDP)	MGC-803	Curcumin: 15 µM. Combination groups: CUR + LD FP = curcumin 15 µM + 5-FU 25 µM + cisplatin 1 µM; CUR + MD FP = curcumin 15 µM + 5-FU 50 µM + cisplatin 2 µM. Comparator FP-alone groups: MD FP = 5-FU 50 µM + cisplatin 2 µM; HD FP = 5-FU 100 µM + cisplatin 4 µM. Viability was assessed at 24, 48, and 72 h; apoptosis/cell-cycle/caspase and Western blot assays at 24 h.	Main Mechanistic Endpoint(s): Apoptosis (Bax, Bcl-2, caspase-3, caspase-8); S-phase arrest; colony formation and migration inhibition. Key Interaction Result: Curcumin enhanced FP activity, reducing viability, colony formation, and migration and increasing apoptosis and S-phase arrest relative to FP-alone comparator groups.	Curcumin potentiated FP-induced anticancer effects in MGC-803 cells. Combined treatment significantly reduced cell viability, colony formation, and migration compared with untreated controls, and enhanced apoptosis, as evidenced by increased Bax, caspase-3, and caspase-8 activities and reduced Bcl-2 expression. At 48 h, CUR + LD FP was more effective than MD FP, and CUR + MD FP was more effective than HD FP in reducing viability. Combination treatment also increased S-phase arrest (38.23% for CUR + LD FP vs. 30.32% for MD FP; 76.38% for CUR + MD FP vs. 47.77% for HD FP), enhanced colony-growth inhibition (38.1% vs. 31.9% and 68.1% vs. 40.0%, respectively), and increased caspase activity (caspase-3: 22.1 vs. 12.5; caspase-8: 20.1 vs. 11.3 for CUR + MD FP vs. HD FP). Curcumin was interpreted as a chemosensitizing/synergistic adjunct to FP chemotherapy, although the reduction in migration was not significantly greater than with FP alone.	Chemosensitization without formal synergy analysis.	[226]
*Allium sativum* L. Amaryllidaceae	crude 65% ethanolic garlic extract	Doxorubicin (DOX)	MKN74	*A. sativum* extract: 0.3 μg/mL; DOX: 1 µM; co-treatment for 24 h	Main Mechanistic Endpoint(s): CDH1 upregulation; COX2 downregulation; viability reduction; metastasis-related marker modulation. Key Interaction Result: Garlic extract enhanced doxorubicin cytotoxicity and reduced viability more than doxorubicin alone.	The Polish *A. sativum* extract increased CDH1 and decreased COX2 expression in MKN74 cells, and showed a synergistic interaction with DOX, reducing cell viability to 52.12 ± 2.83%, compared with 84.03 ± 2.88% for DOX alone. These findings support a chemosensitizing effect of garlic extract in this GC model. The Malaysian *A. sativum* extract upregulated CDH1 and downregulated COX2 in MKN74 cells and exerted a synergistic effect with DOX, reducing cell viability to 62.63 ± 2.31% vs. 84.03 ± 2.88% with DOX alone. Overall, the extract enhanced DOX cytotoxicity and modulated invasion/metastasis-related markers.	Chemosensitization without formal synergy analysis.	[208]
*Glycyrrhiza glabra* L. Fabaceae	Liquiritin (LIQ)	TRAIL (tumor necrosis factor-related apoptosis-inducing ligand; non-conventional antitumor biologic rather than standard chemotherapy)	AGS, SNU-216	In vitro: TRAIL 80 ng/mL + LIQ 50 µM for 24 h in most mechanistic assays; viability studies used LIQ 0–200 µM with/without TRAIL for 24 and 48 h. In vivo: TRAIL 100 μg/mouse/day i.p. + LIQ 20 mg/kg/day i.p. for 28 days in AGS and SNU-216 xenograft-bearing nude mice.	Main Mechanistic Endpoint(s): Apoptosis (caspase-3/-8/-9, PARP cleavage); DR4/DR5/FADD upregulation; ROS/JNK signaling; EMT inhibition; migration and colony suppression. Key Interaction Result: Liquiritin sensitized TRAIL-resistant gastric cancer cells to TRAIL and reduced xenograft growth more than either treatment alone.	LIQ sensitized TRAIL-resistant GC cells to TRAIL, producing a synergistic antitumor effect. Combined treatment reduced viability more strongly than either agent alone, lowered the IC_50_ of LIQ in both AGS and SNU-216 cells, suppressed migration and colony formation, and inhibited EMT by increasing E-cadherin while decreasing N-cadherin, vimentin, and Twist. The combination also enhanced apoptosis through both intrinsic and extrinsic pathways, with activation of caspase-3/-8/-9, PARP cleavage, upregulation of DR4/DR5/FADD, increased ROS production, and JNK phosphorylation; these effects were attenuated by NAC, indicating ROS dependence. In vivo, TRAIL/LIQ significantly reduced xenograft tumor growth, increased TUNEL positivity, and decreased Ki-67 expression.	General anticancer activity without direct evidence of chemotherapy interaction.	[227]
*Camellia sinensis* (L.) Kuntze Theaceae	(−)-Epigallocatechin-3-gallate (EGCG)	5-Fluorouracil (5-FU)	SGC-7901/FU, MGC-803/FU (5-FU-resistant derivatives); in vivo: SGC-7901/FU xenograft	In vitro: EGCG 20 µM for 72 h in proliferation assays; 30 µM for 48 h in MDR-1/P-gp assays; 5-FU sensitivity assessed at 1–200 µM for 72 h. In vivo: EGCG 25 mg/kg + 5-FU 20 mg/kg for 30 days in the xenograft model.	Main Mechanistic Endpoint(s): MDR-1/P-gp down-regulation; TFAP2A/VEGF axis inhibition; proliferation suppression. Key Interaction Result: EGCG reversed 5-FU resistance and enhanced 5-FU antitumor activity in resistant GC models.	EGCG reversed 5-FU resistance and acted as a chemosensitizer in GC models. It inhibited the proliferation of both parental and resistant cells, with a stronger effect in resistant cells, and downregulated MDR-1 and P-gp at both mRNA and protein levels. In resistant xenografts, co-treatment with EGCG + 5-FU more strongly suppressed tumor volume and weight than either agent alone, while also decreasing VEGF and p-TFAP2A, supporting sensitization through inhibition of the TFAP2A/VEGF axis and drug-resistance proteins.	Chemosensitization without formal synergy analysis.	[228]
*Syzygium aromaticum* (L.) Merr. & L.M.Perry Myrtaceae	Eugenol; eugenol-loaded mesoporous silica nanoparticles (eugenol-MSN)	Cisplatin	AGS	Single treatment: cisplatin 0.5–6 μg/mL; free eugenol and eugenol-MSN 1–80 μg/mL for 48 h. Combination treatment: free eugenol or eugenol-MSN 2.5, 5, 10, 20 μg/mL plus cisplatin 0.5–6 μg/mL for 48 h. Migration assay: IC_10_-based treatments for 72 h. Gene-expression assays: IC_30_-based treatments for 48 h.	Main Mechanistic Endpoint(s): Apoptosis (caspase-3, caspase-8, caspase-9); MDM2, MMP9, and KRAS reduction; migration inhibition. Key Interaction Result: Combination treatment was more effective than either agent alone, and free eugenol, especially eugenol-MSN, enhanced cisplatin sensitivity.	Free eugenol, and especially eugenol-MSN, enhanced cisplatin sensitivity in AGS cells, with a synergistic interaction (CI < 1). Combination treatment was more effective than single agents in reducing viability, inhibiting migration, and inducing apoptosis. It also increased the expression of caspase-3, caspase-8, and caspase-9 and significantly decreased the expression of MMP2, MMP9, and KRAS. The authors additionally reported that free eugenol showed stronger DPPH radical-scavenging activity, whereas eugenol-MSN more strongly increased SOD activity.	Formal synergy is demonstrated by quantitative combination analysis.	[229]
*Marsdenia tenacissima* (Roxb.) Wight & Arn. Apocynaceae	*Marsdenia tenacissima* (Roxb.) Wight & Arn. (source of Xiaoaiping (XAP) injection	S-1	BGC-823, MGC-803; in vivo: BGC-823 xenograft model	—	Main Mechanistic Endpoint(s): VEGF, MMP-9, N-cadherin, and vimentin downregulation; E-cadherin upregulation; invasion/migration/adhesion suppression. Key Interaction Result: XAP combined with S-1 enhanced anti-tumor and anti-invasive effects relative to either treatment alone.	XAP combined with S-1 enhanced the antitumor effect versus either agent alone. The combination increased growth inhibition, reduced cell adhesion, migration, and invasion, and modulated metastasis/EMT-related markers, with decreased VEGF, MMP-9, N-cadherin, and vimentin, alongside increased E-cadherin. In vivo, the combination significantly suppressed tumor growth more effectively than XAP or S-1 alone, supporting a synergistic anti-invasive and anti-metastatic effect.	Chemosensitization without formal synergy analysis.	[230]
*Scutellaria baicalensis* Georgi Lamiaceae	Wogonin (WOG)	Paclitaxel (PTX)	BGC-823, MGC-803, HGC-27, MKN-45; in vivo: BGC-823 xenograft	In vitro: cells were exposed to WOG + PTX for 48 h at a fixed ratio based on their respective IC_50_ values. At Fa = 0.5, the combination regimens were: MGC-803: WOG 17.79 µM + PTX 5.93 µM; HGC-27: WOG 13.42 µM + PTX 22.34 µM; MKN-45: WOG 46.87 µM + PTX 2.58 µM. In BGC-823, WOG + PTX was antagonistic at Fa = 0.5 (CI 1.05), although synergism was observed at lower Fa values. In vivo: WOG 60 mg/kg daily + PTX 10 or 20 mg/kg weekly for 2 weeks.	Main Mechanistic Endpoint(s): Growth inhibition; apoptosis enhancement; tumor-weight inhibition in vivo. Key Interaction Result: Wogonin enhanced paclitaxel activity in most tested models and enabled low-dose PTX combinations to achieve substantial tumor inhibition, although the interaction was cell-line- and effect-level-dependent.	WOG enhanced the anticancer activity of PTX in GC. In vitro, the combination showed synergistic growth inhibition in most cell lines within defined Fa ranges and increased apoptosis compared with either agent alone. In vivo, WOG + low-dose PTX (10 mg/kg) achieved markedly greater tumor inhibition than either single treatment, with tumor-weight inhibition rates of 61.58% for the combination vs. 20.29% for WOG alone and 22.27% for low-dose PTX alone. The combination of low-dose PTX achieved efficacy comparable to high-dose PTX, without the apparent toxicity associated with high-dose PTX alone.	Formal synergy is demonstrated by quantitative combination analysis.	[231]

## Data Availability

No new data were created or analyzed in this study. Data sharing does not apply to this article.

## Abbreviations

The following abbreviations are used in this manuscript:

18β-GA	18β-glycyrrhetinic acid
5-FU	5-fluorouracil
AGE	aged garlic extract
BDMC	bisdemethoxycurcumin
CAFs	cancer-associated fibroblasts
CEO	clove essential oil
CSCO	Chinese Society of Clinical Oncology
DADS	diallyl disulfide
DAS	diallyl sulfide
DATS	diallyl trisulfide
DMC	demethoxycurcumin
EC	epicatechin
ECG	epicatechin-3-gallate
EGC	epigallocatechin
EGCG	(−)-epigallocatechin-3-gallate
GC	Gastric cancer
GTE	green tea extract
OSC	organosulfur compound
SAC	S-allyl-L-cysteine
SAMC	S-allylmercaptocysteine
